# How Much Is Enough? An Empirical Test of the Resource Dispersion Hypothesis

**DOI:** 10.1002/ece3.72696

**Published:** 2025-12-17

**Authors:** Sourabh Biswas, Kalyan Ghosh, Sumedha Touhid, Srijaya Nandi, Arpan Bhattacharyya, Arunima Bhattacharyya, Milisha Das, Raktim Paul, Anindita Bhadra

**Affiliations:** ^1^ Behaviour and Ecology Lab, Department of Biological Sciences Indian Institute of Science Education and Research Kolkata Mohanpur West Bengal India; ^2^ Department of Life Sciences Presidency University Kolkata West Bengal India; ^3^ Department of Biotechnology Maulana Abul Kalam Azad University of Technology Nadia West Bengal India; ^4^ Department of Microbiology Kanchrapara College Kanchrapara West Bengal India; ^5^ Department of Microbiology Ramakrishna Mission Vivekananda Centenary College Rahara West Bengal India

**Keywords:** free‐ranging dogs, group size, Resource Dispersion Hypothesis, territory size, urbanisation

## Abstract

Understanding how animals adjust their spatial and social organization to resource variability is central to ecological theory. We tested predictions of the Resource Dispersion Hypothesis (RDH) by examining how resource availability and distribution influence territory and group structure in a species highly adapted to the Anthropocene: the free‐ranging dog ( *Canis lupus familiaris* ). Across rural and urban sites in India, we conducted census‐based surveys and territory‐based tracking over three reproductive seasons to assess spatial and social dynamics along resource gradients. Dog and resource densities were significantly higher in urban areas, but a generalized linear model revealed that resources predicted adult dog density only in rural sites, with this relationship disappearing in urban environments. Territory size varied seasonally, decreasing by 21% post‐mating, and was positively correlated with resource heterogeneity, dispersion, patch richness, and male:female ratio. However, group size remained stable across seasons, suggesting a decoupling of spatial and social responses to resource distribution. These findings support the RDH and highlight the complex interplay between ecological and social factors in shaping animal behaviour within anthropogenic landscapes. The results have implications for urban wildlife management, informing targeted interventions such as waste regulation and sterilisation programmes.

## Graphical abstract

We provide evidence in support of the Resource Dispersion Hypothesis in free‐ranging dogs of India.

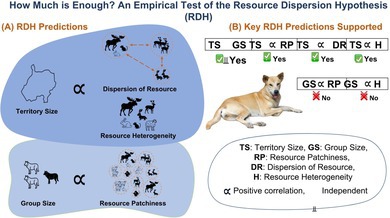

## 1 Introduction

Group living is a widespread behavioural trait observed across animal taxa, from insects to mammals. The structure of these social groups varies widely—ranging from loose aggregations, as seen in many ungulates, to highly organized systems, such as those of eusocial insects. While group living can confer numerous benefits, including predator avoidance, cooperative foraging, and enhanced care of offspring, it also imposes costs. These include intensified competition for resources, increased risk of disease transmission, and unequal fitness returns due to hierarchical social structures. The evolutionary stability of group living is therefore shaped by trade‐offs between these costs and benefits (Pyke 1979).

The Resource Dispersion Hypothesis (RDH) (Macdonald 1983) offers one explanatory framework for the evolution of group living, particularly in species utilizing patchily distributed resources. It posits that territory size is primarily driven by the spatial dispersion of resources, while group size is influenced by resource heterogeneity and richness. Critically, RDH predicts that groups may form even in the absence of social underpinnings, provided territories encompassing essential resource patches can support multiple individuals. While the sustainability of these groups necessitates social interactions, the formation itself may emerge from ecological constraints alone.

Over the past three decades, RDH has received both empirical support and criticism. It was originally developed based on carnivore social behaviour, and studies on species like red foxes ( *Vulpes vulpes* ) (Macdonald 1983; Doncaster and Macdonald 1997) and spotted hyenas ( *Crocuta crocuta* ) (Kruuk 1985) have supported its predictions. However, other studies, such as those on European otters ( *Lutra lutra* ) and wolverines ( *Gulo gulo* ), have recorded deviations from RDH expectations (Kruuk and Hewson 1978; Kruuk et al. 1989; Vangen et al. 2001), highlighting the role of other ecological and social factors, such as competition, predation, and conspecific interactions, in shaping group formation and territory size.

Canids are particularly suitable for testing the RDH, given their wide variation in social organization, from solitary species (Dietz 1984) to pair‐living (Kamler and Macdonald 2014) and highly coordinated packs (Zubiri and Gottelli 1995; Creel et al. 2004). Among them, free‐ranging dogs (FRDs) ( *Canis lupus familiaris* ) occupy a unique niche. Unlike owned pet dogs, FRDs form unrestrained, freely breeding populations in human‐dominated landscapes (Serpell 1995; Bergström et al. 2020). These dogs primarily scavenge from anthropogenic waste, with minimal reliance on hunting, especially in urban environments (Bhadra et al. 2016; Biswas, Bhowmik, et al. 2024; Sen Majumder, Bhadra, et al. 2014). The resources they depend on are not only variable in quality and quantity but are also highly patchy in their spatial distribution.

FRDs often form social groups and display a range of affiliative and competitive behaviours within and between groups. This makes them an excellent model system for studying how resource distribution influences both territoriality and social structure. Urban areas typically present higher resource densities and more predictable food availability due to waste concentration and human provisioning, which may promote smaller territories (Sambo et al. 2018; Thanapongtharm et al. 2021). In contrast, rural areas, with sparser and less predictable resources, may necessitate larger territories to ensure access to food and shelter (Valenzuela and Macdonald 2002; Warret Rodrigues and Roth 2023).

The social structure of FRDs is also expected to respond to resource conditions, albeit in more complex ways. In resource‐rich habitats, larger groups may be ecologically viable but may also face anthropogenic limitations due to human intolerance. In contrast, under resource‐scarce or clumped conditions, individuals may benefit more from solitary living to reduce competition or from group living to enhance foraging efficiency (Travis and Slobodchikoff 1993; Travis et al. 1995). Additionally, the function of grouping may shift seasonally. Previous work has shown that adult dogs form more opposite‐sex pairs during the mating season, while juveniles tend to remain near adults in non‐mating periods, suggesting both reproductive and protective roles for grouping (Sen Majumder, Chatterjee, et al. 2014).

Despite increasing interest in free‐ranging dogs as a study system, there remains a lack of comprehensive understanding of how territory size and group composition vary with seasonal resource dynamics. In this study, we examine how spatial and social patterns in FRDs respond to resource dispersion across urban and rural landscapes in India. Guided by the Resource Dispersion Hypothesis (RDH), we test four linked predictions that distinguish the drivers of spatial and social organization.

First, we predict that territory size will be primarily determined by the spatial dispersion and heterogeneity of resource patches. In line with the RDH, we hypothesize that territory size is set by the minimum area required to encompass the most spread‐out, necessary patches, and thus, territory size will increase with greater spatial dispersion (i.e., larger mean inter‐patch distances) and higher spatial heterogeneity (i.e., greater variance in patch locations).

Second, we predict that group size and composition will be determined by the richness of resources within that territory. Unlike territory size, we propose that group size is set by the within‐territory carrying capacity. We hypothesize that higher mean patch richness (i.e., greater food abundance) will support larger, more complex groups (e.g., multi‐adult groups) without necessitating a larger territory.

Third, as a key corollary of the first two predictions, we predict that territory size and group size will be statistically independent. Because the RDH posits that these two traits are driven by different, decoupled resource characteristics (spatial dispersion and patch richness, respectively), we hypothesize that there will be no necessary association between the area a group defends and the number of individuals within it. This directly contrasts with models where group size drives territory size (e.g., via increased defensive needs).

Finally, we predict that both spatial and social structures will be dynamic, following temporal shifts in resource landscapes. Specifically, territories will expand in seasons when key resources become sparse and more widely dispersed, and contract when resources become predictably aggregated. Concurrently, we predict group size will increase (and fission‐fusion dynamics may attenuate) in seasons characterized by high patch richness, but groups will become smaller or more fluid when resources are poor.

## 2 Methodology

### 2.1 Study Sites and Subjects

#### 2.1.1 Census‐Based Study

**FIGURE A1:**
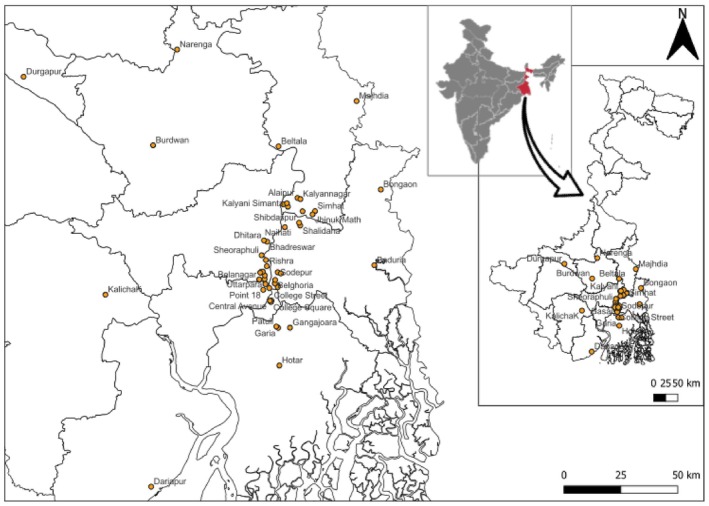
Study sites in West Bengal, India. Orange points mark surveyed locations; inset shows West Bengal within India. Base layers: West Bengal administrative boundary (Eddy, ‘WB shapefile’, Kaggle; accessed 9 August 2024); India outline by फ़िलप्रो (Filpro), CC BY‐SA 4.0 (via Wikimedia Commons; accessed 6 September 2025). Map created in QGIS 3.36; projection WGS84 (EPSG:4326). Scale bars and north arrow shown.

We conducted this census‐based study to investigate the relationship between resource availability and the spatial ecology of free‐ranging dogs. The study was carried out across multiple districts of West Bengal, India, including the Kolkata metropolitan region and the adjacent districts of Howrah, Hooghly, North 24 Parganas, Nadia, Paschim Bardhaman, and South 24 Parganas (Figure A1; Table S1). The study sites were stratified as urban (municipality/corporation towns) or rural (gram‐panchayat villages). This classification was based on the criteria defined by the EAC‐PM, Govt. of India (Ravi 2023), ensuring a standardized and consistent system. We used a stratified spatial approach for site selection, designed to cover the urban–rural gradient while limiting spatial clustering. Within each stratum, we identified candidate towns/villages from administrative maps and prior ground knowledge. The final sites were chosen to ensure geographic spread (e.g., along major transport corridors like the Hooghly riverine towns) and to avoid immediately adjacent areas to reduce spatial dependence.

A total of 93 sites were surveyed (52 rural, 41 urban). The spatial distribution of all sites is shown in Figure A1, and a complete registry of site names and coordinates is provided in Table S1.

##### 2.1.1.1 Site Delineation and Standardization

Within each selected location, a survey polygon (see Figures S1 and S2) was purposefully and manually drawn on Google My Maps to delineate the boundaries of the target human settlement and its adjoining accessible areas (e.g., streets, open spaces). This sampling strategy focused our survey on free‐ranging dogs living in close proximity to human habitation. The aim was to cover an area on foot over a period of 2 h to record the data during the census. Polygon sizes varied (1.29–161 ha), reflecting the inherent difference in settlement density and spatial extent between rural and urban environments. To ensure comparability across these different areas, all variables (e.g., dog counts, resource points) were standardized by polygon area (calculated as units per hectare) prior to analysis.

##### 2.1.1.2 Survey Protocol and Timing

We followed the established spot census method (Sen Majumder, Chatterjee, et al. 2014). Each specified polygon was surveyed once. Surveys were conducted across two periods: June–July 2023 and March–May 2024.

The dog census was strictly conducted between 16:00 and 18:00 h, as this time period is when dogs are typically most active and visible (Sen Majumder, Chatterjee, et al. 2014), facilitating accurate observation. During this census, an observer covered all accessible roads and alleys within the polygon. To avoid re‐sampling, each path was traversed only once.

The resource mapping and household surveys were conducted separately and were not restricted to this time window, as we did not observe significant temporal variation in the availability of these resource points.

##### 2.1.1.3 Dog Census Parameters

During the dog census, several parameters were documented on paper for every free‐ranging dog observed: time of sighting, sex (determined by observing the genitalia), age class (categorized as pup, juvenile, or adult based on body size and genital structures), and social status (solitary or in a group). For dogs in a group, the total number of dogs was recorded, with a group defined as two or more dogs engaging in affiliative interactions (e.g., allogrooming, playing, walking together) or resting peacefully within approximately 1.5 m (~2 body lengths) of each other. Dogs observed in non‐affiliative or aggressive interactions were recorded as separate individuals.

##### 2.1.1.4 Observer Training and Inter‐Observer Reliability

A total of four observers conducted the census surveys. All observers underwent a mandatory 20 h of training, which involved ad libitum sampling on dogs, constructing an ethogram from their own data, and subsequent familiarization with the standardized project ethogram and sampling methods. Following this, all observers conducted three practice sampling sessions in the field with the lead author to ensure accuracy in identifying dogs and resource points. Data collection began only after high inter‐observer consistency was established.

##### 2.1.1.5 Methodological Limitations and Ethical Considerations

We acknowledge that standardizing counts by total polygon area (dogs/ha) when surveys were restricted to accessible roads/alleys could be a limitation. This might result in an underestimation of density in polygons with large, inaccessible private areas. However, we contend that these accessible routes represent the primary foraging and social habitat for free‐ranging dogs in this system, making this density metric a valid and comparable proxy.

While our training protocol was designed to minimize variation, we cannot completely rule out potential biases in dog observations due to minor remaining inter‐observer variability or differences in dog detectability across habitats. For the household survey, a formal response rate was not calculated, but the majority of households approached did consent to participate.

All study procedures were conducted with the utmost respect for the welfare of the dogs and the privacy of residents. Observers maintained a safe distance to minimize disturbance; no dogs were harmed or captured, and informed verbal consent was obtained from households prior to conducting surveys.

#### 2.1.2 Territory‐Based Study

We conducted a long‐term observation‐based study on 36 dog groups from 2020 to 2024 in order to build a comprehensive understanding of the spatial and temporal distribution and territory of FRDs. This study was conducted in urban and suburban areas of Bongaon (5), Gayeshpur (9), Kalyani (4), Bardhaman town (14) and Narenga (4) of West Bengal, India. Observations were carried out across three distinct seasons: the pre‐mating season (April to early July), the mating season (mid‐July to mid‐October), and the post‐mating pup emergence season (Late October to March). These seasons were chosen to capture the dynamic interplay between resource fluctuation, territory size, and social behaviour in free‐ranging dogs. Some of these sites were adjacent to areas with seasonal fairs or events that temporarily increase resource abundance (Biswas, Ghosh, et al. 2024).

We defined a group as a set of free‐ranging dogs observed repeatedly in the same area, displaying affiliative interactions among themselves and maintaining a common territory. Each member of the group was given a unique identity based on their sex and morphological features. At each visit, we recorded the individuals present and membership status (resident vs. transient), noting join/leave events (e.g., pup additions post‐mating with high early‐life mortality; adult deaths or immigration/emigration, especially around the mating season). Analyses of seasonal change in group properties (e.g., territory size) were therefore based on the resident subset.

In this study, a territory was defined as an area that an animal or group of animals consistently defended against conspecifics (and occasionally other animals) to secure access to resources and mates. To determine territory boundaries, we conducted behavioural observations of at least 30 h per group per season, following an established protocol (Paul et al. 2014a) that used randomized instantaneous scans (SCAN) and all occurrences sessions (AOS).

Observation sessions, each lasting 3 h, were distributed across four time intervals to ensure full daylight coverage: early morning (6:00 a.m. to 9:00 a.m.), morning (9:00 a.m. to 12:00 p.m.), noon (12:00 p.m. to 3:00 p.m.), and afternoon (3:00 p.m. to 6:00 p.m.).

**FIGURE 1:**
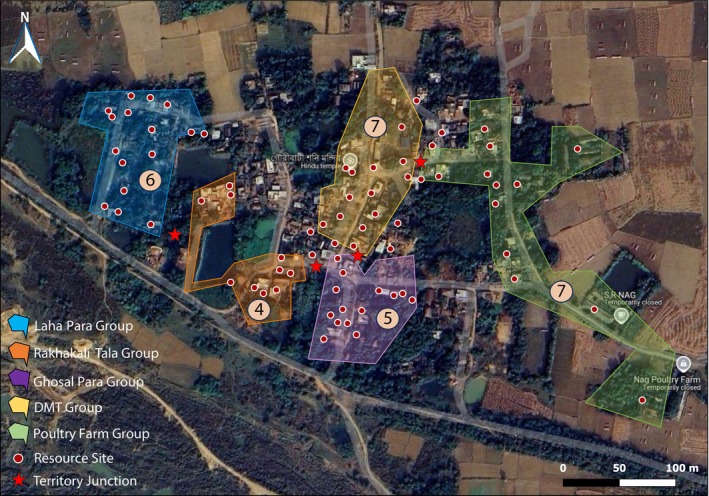
Map of the study site in Narenga, Purba Bardhaman, West Bengal, India, showing the territories of five free‐ranging dog groups. The coloured polygons represent the respective territories: Laha Para Group (blue), Rakhakali Tala Group (orange), Ghosal Para Group (purple), DMT Group (yellow), and Poultry Farm Group (green). The number inside the beige circle in each polygon indicates the number of resident dogs in that territory. Red dots represent resource sites, and red stars mark territory junctions. Basemap imported from Google My Maps (Google, 2025); layers compiled and styled in QGIS 3.36 with projection WGS84 (EPSG:4326). Scale bar and north arrow shown.

**FIGURE A2:**
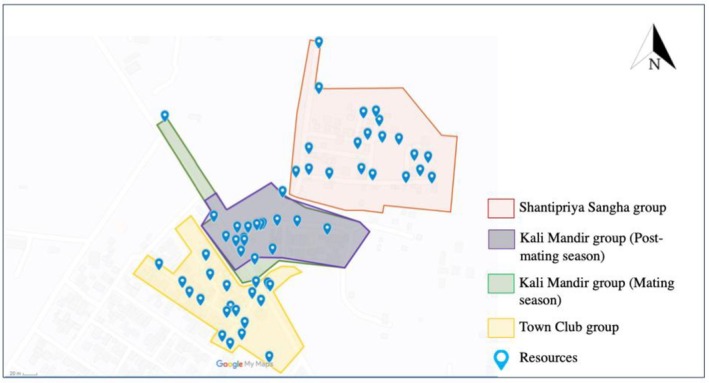
Coloured polygons represent the respective territories of each group. The light green polygon depicts the territory of one group before the mating season. This territory changed after the mating season, and its post‐mating boundary is shown by the violet polygon. The territories of the other two groups remained unchanged throughout the study period. Blue balloons indicate resource points within the territories. © Google My Maps 2025.

Within each 3‐h session, we followed a repeating, randomized protocol. This protocol consisted of either a 5‐min AOS (during which detailed behavioural data were recorded) or a 1‐min SCAN observation. Each observation period, whether AOS or SCAN, was immediately followed by a 2‐min break. This observation‐break cycle was repeated throughout the 3‐h session, yielding a total of 18 scans and 18 AOSs per session (Paul et al. 2014a). During these observations, we recorded instances of aggressive interactions with adjacent groups, noting the sites of urine marking by individual dogs and the spots actively defended during territorial fights, using GPS. These points were then plotted on Google My Maps and connected to create polygons representing the territories (Figure 1; Figure A2). While some degree of territory overlap was observed, particularly among related groups, the core areas of exclusive use were clearly unique for each group.

### 2.2 Statistical Analysis

All statistical analyses were conducted in R Studio (version 4.2.0) (R Core Team 2024).

All the GLMs and GLMMs were performed using ‘lme4’ package (Bates et al. 2015) and ‘glmmTMB’ package (Bolker 2019) in R. The final models were selected based on the lowest AIC value. The null and observed models were compared for the models. Model diagnostics were checked using the ‘performance’ package (Lüdecke et al. 2021) and ‘DHARMa’ package (Hartig and Hartig 2022) of R.

Resources within each territory were surveyed in the same manner as in the census‐based study, ensuring consistency in resource assessment across both components of the research.

To minimize observer effects on dog behaviour and territorial interactions, we maintained a safe distance from the dogs throughout the observation period and avoided any direct interaction with them.

#### 2.2.1 Census‐Based Study

No a priori power calculation was performed for this study. We therefore report a sensitivity analysis for the urban–rural contrasts given the realized sample sizes (*n* = 52 vs. 41, *α* = 0.05, two‐sided): power is ~0.81 for Cohen's SD = 0.6. Using the normal‐shift equivalence, this corresponds to ~80% power at Mann–Whitney AUC ≈ 0.662 (Cliff's *δ* ≈ 0.324) using ‘pwr’ package (Champely et al. 2020).

Dog density was quantified as the number of dogs observed within each survey polygon divided by its area (dogs ha^−1^). Resource density was computed as the area‐standardized sum of resource scores: each resource feature was assigned a score *sj* (by type; see Table S1 for details), the scores were summed within the polygon, and the total was divided by polygon area (∑*j sj*)/*A* to yield resource density in ‘resource ha^−1^’.

Dog and resource densities between rural and urban sites were compared using a two‐sided Mann–Whitney *U* (Wilcoxon rank‐sum) test. We modelled dog density (dogs ha^−1^) as a function of resource density (resource ha^−1^), settlement type (urban/rural), and their interaction term using a generalized linear model (GLM) with a Gamma distribution and log link function (i.e., dogs ha^−1^ ~ resource ha^−1^ × settlement type). Model diagnostics (residuals, dispersion) supported the Gamma–log specification.

Differences in dog density between sexes within urban and rural subsets were assessed using a Wilcoxon rank‐sum test with continuity correction.

#### 2.2.2 Territory‐Based Study

##### 2.2.2.1 Quantification of Resource Availability

We quantified resource availability using two methods. First, a direct visual census was conducted where the observer walked the entire polygon and marked the GPS positions of all resources accessible to dogs (Bhattacharjee and Bhadra 2021). These resource points (detailed in Table 1) included public sources (e.g., waste bins, meat shops, markets) and private anthropogenic sources (e.g., direct feeding by households, eateries).

**TABLE 1:** This table presents a scoring system for food sources used by free‐ranging dogs.

S. No.	Food source	Description	Score
1	Meat shop or fish shop	Open‐air shops only, dogs get access to meat or fish scraps	8
2	Eatery/restaurant/hostel	Roadside open‐air restaurants/eateries and hostels. Restaurants like ‘Subway’ and ‘McDonald's’ were not considered, as free‐ranging dogs do not get access to their leftover food	7
3	Direct feeding by human or NGO	Direct provision of food by humans (may or may not involve begging) from households, or by an NGO/person who feeds a substantial amount to the dogs	6
4	Open garbage dump or occasional carcass	≥ 2 m^2^ in size, consisting of wet and dry garbage, including leftover food by humans. It can accommodate several individuals or rare or occasional carcass of poultry farm or dead animals	5
5	Tea shop or temple	Open‐air shops only. Tea shops may also offer bread/biscuits. Temple: Occasionally on festivals	4
6	Direct feeding by human	Occasional provisioning of food by humans (only by means of begging)	3
7	Grocery/sweet shop/bakery	All these shops come under one umbrella as they offer direct food provisioning to dogs occasionally	2
8	Household or eatery dustbins (without cover)	≤ 0.5 m^2^ in size, consisting of wet and dry garbage. It can accommodate up to two individuals at any given time	1

*Note:* The ‘Score’ column indicates the quality and quantity of available food, with higher scores representing greater access and nutritional value. Descriptions detail the characteristics of each food source.

Second, to quantify the anthropogenic food subsidies (Bhattacharjee and Bhadra 2021), households within each polygon were surveyed (after receiving verbal consent) to assess the frequency and type of food provided. Following the methodology (Bhattacharjee and Bhadra 2021), a score (ranging from 1 to 8) representing food quality and quantity was assigned to each identified resource point based on its type (see Table 1 for full scoring details).

All resource points within each study territory were identified, categorized, and scored (Table 1 following the protocol in Bhattacharjee and Bhadra 2021). While resources encompass food, water, and shelter, this analysis focuses solely on food resources due to data limitations and the nature of water sources in the study area.

We quantified resource distribution using three key metrics:

- *Patch richness*: Calculated as the total number of food resource points available within each territory per season.
- *Resource heterogeneity*: Resource heterogeneity was operationally defined as the richness of available food sources, quantified by a simple count of the distinct food‐source categories present within each territory polygon. To obtain this count, we surveyed each territory during the census for the presence or absence of all categories detailed in Table 1 (e.g., household waste points, community bins/vats, street food stalls, carcass/offal sites, religious offerings, etc.). Therefore, a higher value indicates a broader mix of available food sources. This method adapts an existing resource assessment framework (Bhattacharjee and Bhadra 2021) to our territory‐level mapping.
- *Resource dispersion*: To calculate this, we first determined the distance of each resource point from all other resource points within the territory using the ‘spatstat’ (Baddeley and Turner 2005), ‘sf’ (Pebesma and Bivand 2023) and ‘sp’ (Bivand et al. 2013) packages in R. For each individual resource point, we then calculated an average dispersion score by dividing the sum of distances from that point to all other resource points by the total number of resource points used in the calculation. This resulted in an average dispersion score for each resource point, representing its average distance from all other resources in the territory. These individual resource point dispersion scores were then averaged to obtain the overall resource dispersion score for each territory per season.

We analyzed two responses with Gamma distributions and a log link: (i) territory size in hectares and (ii) total number of adult dogs per territory. For both models, candidate covariates were resource heterogeneity, patch richness, resource dispersion, and male:female ratio. For the territory model, we additionally included the total number of adult dogs; for the dog model, territory size was entered as a predictor. Collinearity was checked (VIFs all low), residuals were examined with DHARMa, and likelihood‐ratio (LR) tests were used to compare each model to its intercept‐only counterpart. To screen for nonlinearity, we fitted generalized additive models (GAMs; Gamma–log) with thin‐plate/cubic regression splines, using REML penalization and term selection implemented in the ‘mgcv’ package (Wood and Wood 2015). Model comparison used AIC and AICc; we report pseudo‐*R* ^2^ (McFadden, Cox–Snell/ML, and Nagelkerke) for GLMs (see Supporting Information for details).

To evaluate the effect of season on territory size, a generalized linear mixed model (GLMM) with a Gamma distribution and log link function was used, incorporating dog group as a random effect. Another GLMM with a Gamma distribution and log link function assessed the effects of season on the total number of adult dogs in a territory, also including dog group as a random effect and using the ‘glmmTMB’ package (Bolker 2019) in R. Model diagnostics were checked using the ‘performance’ and ‘DHARMa’ packages (Hartig and Hartig 2022) in R.

We analysed three resource metrics as separate responses, with season as a fixed effect and group identity as a random intercept where supported by the data. For strictly positive, right‐skewed outcomes (resource heterogeneity, resource patchiness), we used Gamma GLMMs with a log link (glmmTMB). For resource heterogeneity, we compared a constant‐dispersion model against a season‐specific dispersion model (dispersion ~ season) using a likelihood‐ratio test (LRT) and AIC/BIC. For resource patchiness, we retained the standard constant‐dispersion Gamma GLMM. For the average inter‐patch distance (resource dispersion), attempts to fit random‐intercept models were nearly singular/non‐convergent; we therefore modelled log (average inter‐patch distance) using a simple linear model (LM) with season as the only predictor. Model diagnostics were inspected using DHARMa residual simulations for GLMMs and standard residual plots for the LM.

An alpha level of 0.05 was maintained throughout the analysis.

## 3 Results

### 3.1 Census‐Based Study

**FIGURE 2:**
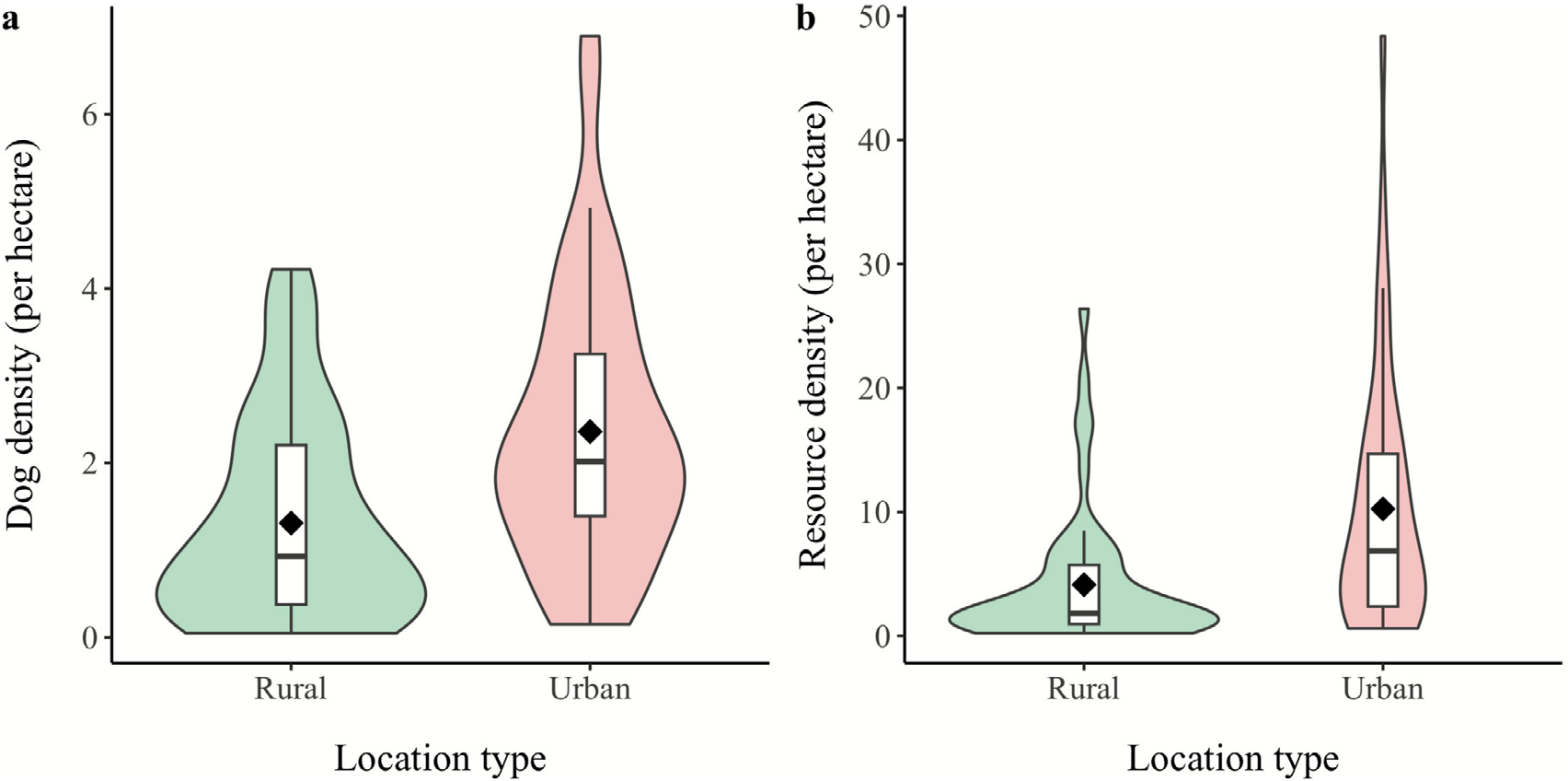
Dog and resource density across rural and urban locations. (a) Dog density (individuals per hectare) and (b) resource density (resource score per hectare) in rural and urban locations. Violin plots with embedded boxplots illustrate the distribution of densities in each location. Black diamonds within the boxplots represent the mean density for each location.

Dog density was higher in urban than rural areas (urban: 2.36 ± 1.57 individuals ha^−1^; rural: 1.31 ± 1.17 individuals ha^−1^; Mann–Whitney *U*: *W* = 620, *p* < 0.001; Figure 2a). Resource density showed a similar pattern (urban: 10.25 ± 10.34 resource score ha^−1^; rural: 4.13 ± 5.53 resource score ha^−1^; Mann–Whitney *U*: *W* = 561, *p* < 0.0001; Figure 2b).

The generalized linear model (GLM) with a Gamma distribution (log‐link) revealed a significant interaction between resource density and location type (*t* = −4.886, *p* < 0.0001), indicating that the effect of resources on dog density fundamentally differs between urban and rural environments (Table 2). In *Rural sites*: We found a strong, positive association. For every one‐unit increase in resource density, dog density increased significantly (estimate = 0.109, *t* = 5.871, *p* < 0.0001). In *Urban sites*: This relationship disappeared. The association between resource density and dog density was effectively flat and not significant (combined slope = 0.003, *p* > 0.05).

**TABLE 2:** Association between dog density (per hectare) and resource density (per hectare).

Term	Estimate	SE	*t* value	*p*	Rate_ratio	RR_CI_ (low)	RR_CI_ (high)
(Intercept)	−0.346	0.128	−2.705	0.008***	0.707	0.55	0.909
Resource ha^−1^ (rural slope)	0.110	0.019	5.871	< 0.0001***	1.116	1.076	1.157
Urban (vs rural)	1.169	0.206	5.684	< 0.0001***	3.219	2.151	4.817
Resource ha^−1^* Urban	−0.106	0.022	−4.886	< 0.0001***	0.899	0.862	0.938

*Note:* GLM formula = dog density per hectare ~ resource density per hectare × settlement type (urban/rural). Asterisks represent the significant path (*p < 0.05; **p < 0.01; ***p < 0.001).

We found no significant difference between the density of male and female dogs in either urban (Wilcoxon rank‐sum test: *W* = 701, *p* = 0.34) or rural (*W* = 398, *p* = 0.25) areas.

### 3.2 Territory‐Based Study

#### 3.2.1 Territory Size and Seasonality

**FIGURE A3:**
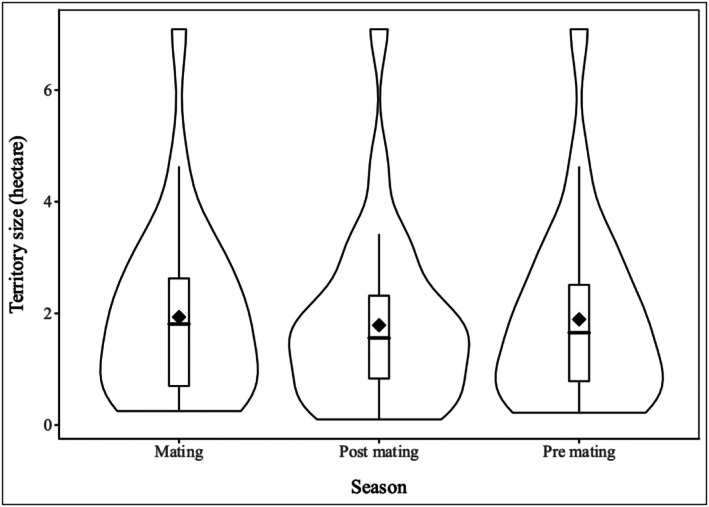
Seasonal variation in dog group territory size. The territory size (hectares) of dog groups across three distinct reproductive seasons: pre‐mating, mating, and post‐mating. Violin plots with embedded boxplots illustrate the distribution of territory sizes in each season. Black diamonds within the boxplots represent the mean territory size for each season.

Territory size, which ranged from 0.101 to 7.909 ha (mean = 1.86 ± 1.66 ha), was found to be dynamic across seasons. A GLMM analysis revealed significant variation in territory size across the three seasons (Figure A3; Table A1). While territory sizes during the pre‐mating (1.89 ± 1.67 ha) and mating (1.93 ± 1.6 ha) seasons were comparable (*p* = 0.48), a significant reduction was observed in the post‐mating season (1.79 ± 1.57 ha; *z* = −2.599, *p* < 0.01) compared to the mating season.

#### 3.2.2 Predictors of Territory Size

**FIGURE 3:**
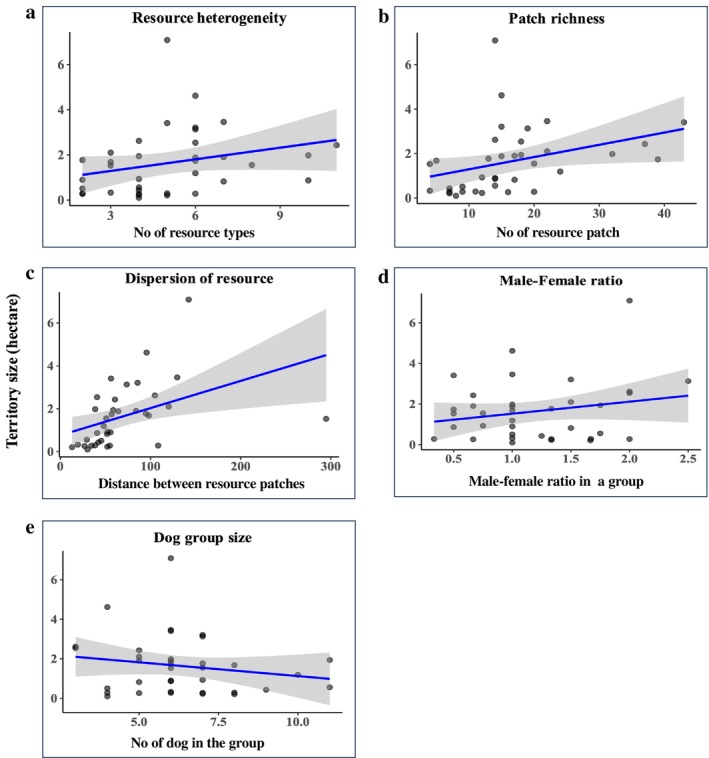
Factors influencing dog group territory size. This figure presents five scatter plots examining the relationship between territory size (hectares) and various ecological predictors. Each black dot represents an individual dog group. The subplots show the relationship with: (a) resource heterogeneity, (b) patch richness, (c) resource dispersion, (d) male–female ratio, and (e) the number of dogs in the group. The solid blue line in each plot represents the predicted mean relationship for that specific variable, as derived from the full GLM (Territory Size ~ Group Size + Resource Heterogeneity + Patch Richness + Resource Dispersion + Male–Female Ratio), holding all other variables constant at their mean. The shaded grey area depicts the 95% confidence interval around this predicted line.

A GLM (Gamma–log) explained territory size well relative to a null model (LR test: Δ Deviance = 18.60 on 5 df, *p* < 0.0001). Further investigation into the factors influencing territory size revealed that resource heterogeneity (*t* = 2.076, *p* < 0.05), patch richness (*t* = 3.271, *p* < 0.01), and resource dispersion (*t* = 6.740, *p* < 0.0001) were all positively associated with territory size (GLM analysis, Figure 3; Table 3). Additionally, the male–female ratio within a group also showed a positive association with territory size (*t* = 2.085, *p* < 0.05). The total number of adult dogs was not a significant predictor of territory size in our models (*p* = 0.99); given the observational design, we do not infer causality.

**TABLE 3:** Results of a generalized linear model (GLM) predicting territory size in free‐ranging dogs.

Term	Estimate	SE	*t* value	*p*
(Intercept)	−2.788	0.514	−5.422	< 0.001***
No of dogs	−0.0006	0.050	−0.011	0.991
Heterogeneity	0.102	0.049	2.076	0.047*
Patch richness	0.041	0.0126	3.271	0.0028**
Male–female ratio	0.391	0.188	2.085	0.046*
Resource dispersion	0.0206	0.0031	6.740	< 0.001***

*Note:* The model examines the influence of group size, resource heterogeneity, patch richness, resource dispersion, and male–female ratio on the size of dog territories. GLM formula: Territory Size ~ Group Size + Resource Heterogeneity + Patch Richness + Resource Dispersion + Male–Female Ratio. Asterisks represent the significant path (*p < 0.05; **p < 0.01; ***p < 0.001).

As a robustness check for potential nonlinearity, we screened with a GAM (Gamma–log). Allowing a smooth only for resource dispersion yielded a small AIC improvement and mild curvature; substantive inferences (direction/significance) were unchanged. We therefore report GLM coefficients as the primary result and provide GAM diagnostics as Supporting Information.

#### 3.2.3 Adult Dogs per Territory

Group size (adults + pups) typically ranged from 3 to 12 individuals (median = 6, mean ± SD = 7.0 ± 4.0, overall range = 3–37). In one season, we observed a single unusually large group of 37 dogs, including 29 pups.

**FIGURE 4:**
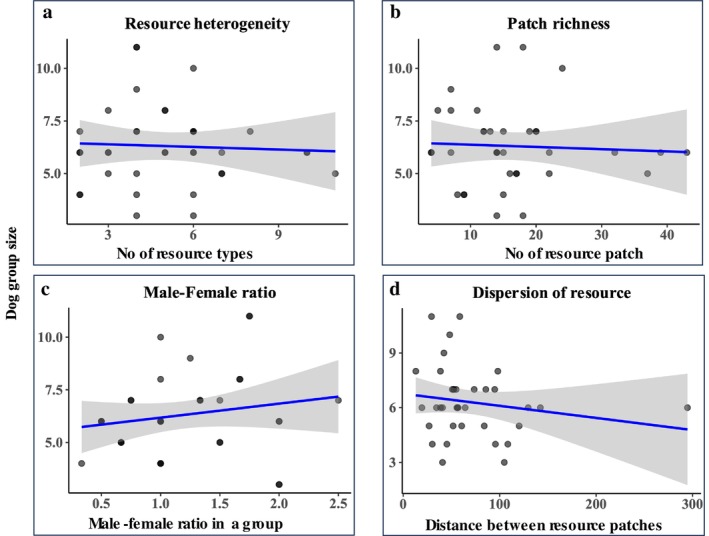
Factors influencing dog group size. This figure presents four scatter plots examining the relationship between group size and various ecological factors: (a) resource heterogeneity, (b) patch richness, (c) male–female ratio within the group and (d) resource dispersion. The solid blue line in each plot represents the predicted mean relationship for that specific variable, as derived from the full GLM (Group Size ~ Resource Heterogeneity + Patch Richness + Resource Dispersion + Male–Female Ratio + Territory size), holding all other variables constant at their mean. The shaded grey area depicts the 95% confidence interval around this predicted line.

Season did not affect the number of adult dogs per territory (GLMM; all seasonal contrasts *p* > 0.05; Table A2). A GLM (Gamma–log) including resource heterogeneity, patch richness, resource dispersion, male:female ratio, and territory size did not improve over the intercept‐only model (LR test: Δ Deviance = 0.305 on 5 df, *p* = 0.646), indicating no detectable joint effect of these covariates on adult counts in this dataset (Figure 4; Table 4). For completeness, we also explored a penalized GAM with small spline bases; this improved AIC via regularization but revealed only weak/near‐linear partial effects. Taken together, we find no strong evidence that resource availability or dispersion metrics predict adult‐dog counts at the territory scale.

**TABLE 4:** Results of a generalized linear model (GLM) predicting group size in free‐ranging dogs.

Term	Estimate	SE	*t* value	*p*
(Intercept)	1.776	0.281	6.313	< 0.001***
Heterogeneity	−0.003	0.029	−0.106	0.916
Patch richness	0.002	0.007	0.389	0.700
Male–female ratio	0.146	0.114	1.279	0.211
Resource dispersion	0.001	0.002	−0.656	0.517
Territory size	−0.027	0.060	−0.451	0.655

*Note:* The model examines the influence of resource heterogeneity, patch richness, resource dispersion, and male–female ratio on the size of dog group. GLM formula: Group Size ~ Resource Heterogeneity + Patch Richness + Resource Dispersion + Male–Female Ratio + Territory Size. Asterisks represent significant path (*p < 0.05; **p < 0.01; ***p < 0.001).

#### 3.2.4 Association With Resource Matrices and Season

##### 3.2.4.1 Resource Heterogeneity

Allowing season‐specific dispersion markedly improved fit over constant dispersion (AIC 212.1 vs. 246.4; LRT *χ* ^2^ = 38.29, df = 2, *p* < 0.0001; *N* = 84, 36 groups). Mean seasonal effects were small and non‐significant (post‐mating: −0.031 ± 0.025, *p* = 0.216; pre‐mating: 0.008 ± 0.008, *p* = 0.288). Group‐level variance was modest (Var = 0.178, SD = 0.423). The dispersion model indicated lower dispersion in post‐mating (−2.648 ± 1.102, *p* = 0.016), with no difference in pre‐mating (1.438 ± 6.549, *p* = 0.826). DHARMa checks were unproblematic.

##### 3.2.4.2 Resource Patchiness

The Gamma GLMM (log link) showed no seasonal differences (post‐mating: −0.019 ± 0.021, *p* = 0.369; pre‐mating: 0.002 ± 0.022, *p* = 0.920). Residual simulations were acceptable.

##### 3.2.4.3 Resource Dispersion

Mixed‐effects variants were near‐singular, so we report a simple LM on log: no seasonal effect [*F*_(2,81)_ = 0.559, *p* = 0.574; *R* ^2^ = 0.0136; *N* = 84]. Estimates were small and non‐significant (post‐mating: −0.118 ± 0.151, *p* = 0.434; pre‐mating: −0.169 ± 0.168, *p* = 0.319). Diagnostics were satisfactory.

For details of the results of association with resource matrices and season, see Supporting Information.

## 4 Discussion

**FIGURE 5:**
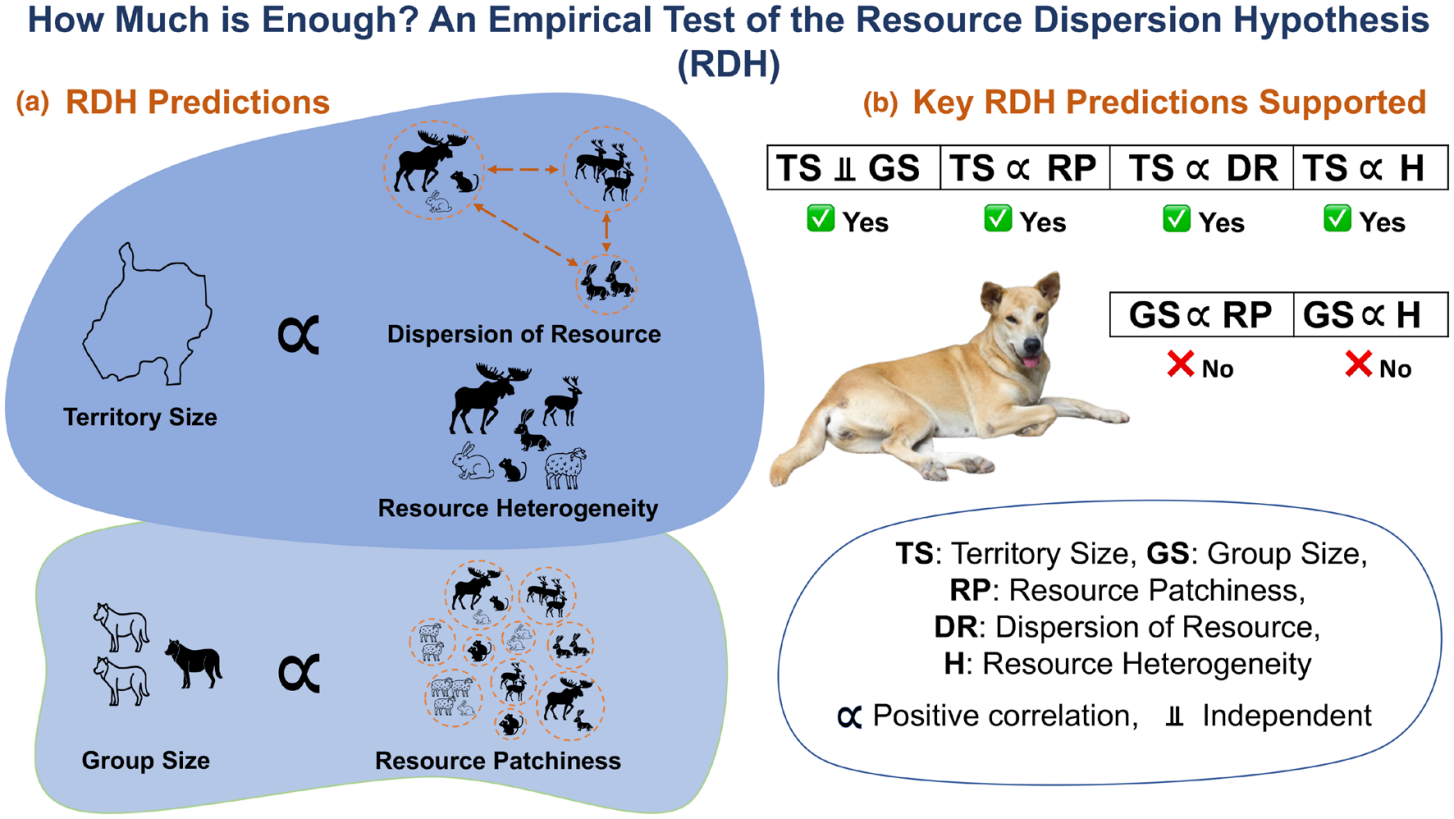
Infographic summarizing the results of our study in the light of the Resource Dispersion Hypothesis (RDH). (a) Illustrates key predictions of the RDH: Territory size (TS) is expected to increase with both resource dispersion (DR) and resource heterogeneity (H), while group size (GS) within a territory is predicted to correlate with resource patchiness (RP) but remains independent of TS. (b) Summarizes our empirical findings, which support these RDH predictions. We observed positive associations between TS and both DR and H, and between GS and RP, while GS and TS were independent—aligning with RDH expectations.

Free‐ranging dogs are an excellent model system for studying animal adaptation to human‐dominated landscapes and can provide insights into managing the rapidly urbanizing habitats for better co‐existence between humans and other species. Our study, conducted across multiple rural and urban sites, aimed at testing predictions of the Resource Dispersion Hypothesis, revealed that across the urban–rural gradient, dog density relates to resources differently in cities versus the countryside. Although both dog and resource densities were higher in urban sites, resources predicted dog density only in rural sites, whereas the relationship flattened in urban sites. At the territory scale, space use increased with resource patch richness and inter‐patch distance (resource dispersion) and with higher male:female ratios, while resource heterogeneity (number of food‐source categories) had a weaker positive effect. Territories contracted in the post‐mating season, consistent with reduced ranging during pup care. Together, these results indicate that the configuration of anthropogenic resources—richness and spacing more than raw counts—shapes where dogs range, while local adult counts are not straightforwardly explained by the same metrics at this scale (see Figure 5 for a synopsis).

Free‐ranging dogs are obligate scavengers (Sarkar et al. 2019) strongly impacted by anthropogenic activities, as their resource landscape is structured by human‐generated waste and deliberate provisioning (Sen Majumder, Bhadra, et al. 2014) rather than natural prey. Prior work documents their flexibility in social organization and reliance on anthropogenic food sources (Biswas, Ghosh, et al. 2024) and the present study shows that spatial features of the anthropogenic resource mosaic (patch richness, dispersion, category diversity) translate into territory size, but these features do not directly predict adult counts at the territory scale.

At the habitat (census) scale, cities had higher average dog density and higher resource density than rural areas, with the resource–density relationship being habitat‐dependent. In rural sites, dog density rose steeply with resource density (GLM Gamma–log; slope ≈ 0.109), supporting RDH expectations that local abundance impacts resource availability (Macdonald 1983). In urban sites, the relationship flattened (combined slope ≈ 0.003), despite higher mean levels of both dogs and resources. This urban decoupling implies that additional urban‐specific forces like routine human provisioning, refuse management, conflict/sterilization pressure, space use constraints, or saturation effects dampen the marginal effect of extra resources on abundance (Sambo et al. 2018; Thanapongtharm et al. 2021). Thus, while rural dog density appears resource‐limited in the RDH sense, urban dog density is multi‐causal, with resources being a necessary but not sufficient condition to explain the observations. This suggests that other anthropogenic activities like vehicular traffic, built‐up space, human responses to dogs, etc. are likely to influence the distribution of dogs. Further studies can be conducted to understand the impact of human behaviour on dog densities and distribution in urban habitats.

These population‐level results dovetail with our territory‐scale findings: the configuration of anthropogenic resources (patch richness and spacing) shaped space use, whereas simple counts were weaker predictors. Together, they suggest (i) at broad scales, mean urban abundance is high but not linearly driven by marginal resource increases; and (ii) at fine scales, how resources are arranged governs where dogs range. This aligns with the species' obligate scavenging and tight coupling to human activity (Sarkar and Bhadra 2022), their behavioural flexibility and reliance on anthropogenic food (Biswas, Ghosh, Gope, and Bhadra 2025), and reports of high urban densities moderated by human mediation (Sambo et al. 2018; Thanapongtharm et al. 2021). The lack of sex differences across habitats indicates that resource landscapes influence total abundance rather than sex structure, consistent with prior work on free‐ranging dog population variation (Warembourg et al. 2021).

Moving beyond population‐level patterns, we investigated the influence of resource dispersion on individual space use by examining territory size (TS) across seasons and in relation to resource attributes. Our findings demonstrate that TS is quite dynamic; it decreases during the post‐mating season as compared to the mating season, while remaining relatively stable in the pre‐mating period. This seasonal variation in TS likely reflects shifts in resource demands and distribution, potentially linked to breeding activities and the energetic costs of reproduction (Marneweck et al. 2019; Jenner et al. 2011), as well as mating interests.

However, this dynamic is further complicated by the unique fluctuations of human‐generated food waste (Bhattacharjee and Bhadra 2021), a key resource for free‐ranging dogs. Periods of abundance, perhaps following festivals or holidays, might allow dogs to contract their territories, capitalizing on readily available food (Biswas, Ghosh, et al. 2024). Conversely, leaner times could necessitate expanding their ranges to encompass a wider array of potential food sources. This flexibility in space use underscores their adaptability and resilience in a fluctuating resource landscape.

Our results further support the Resource Dispersion Hypothesis (RDH), indicating that territory size (TS) is positively correlated with resource heterogeneity, dispersion, and patch richness, but not with group size. This suggests that while dogs may expand their territories to encompass a wider array of resources, the sizes of their social groups remain stable regardless of resource availability. This suggests an adaptive understanding of the spatial distribution of human‐derived resources. Free‐ranging dogs are not simply responding to overall abundance, but also to the spatial configuration of these resources, demonstrating a capacity for sophisticated spatial memory and decision‐making within the human‐modified environment. As resource patches become more dispersed, individuals may need to increase their TS to secure sufficient resources, but this expansion does not necessarily translate to larger group sizes (Erlandsson et al. 2022; Valeix et al. 2012). This decoupling of TS and group size is a crucial observation and aligns with the predictions of the RDH, as well as empirical findings in other social carnivores, such as wolves (Kittle et al. 2015). In fact, we speculate that in areas where a large number of dogs are provided ample food in bulk by dedicated human feeders, the territory sizes might be much reduced, leading to a high density of dogs in such pockets. Future studies need to be conducted to test this prediction.

The positive association between TS and male‐to‐female ratio suggests that increased male–male competition for mates is another key factor influencing territory size. This could be due to males needing to cover more ground to find mates (Clutton‐Brock 1995), defend resource‐rich areas that attract females (Alatalo et al. 1986), or engage in more aggressive territorial defence (David 1970; Mitani and Rodman 1979). For instance, studies in red deer have demonstrated that males with larger territories have greater mating success (Clutton‐Brock et al. 1982). However, a key limitation of our study is that territories were defined at the group scale. This prevented us from testing for sex‐specific differences in individual home‐range sizes. Therefore, the observed association must be interpreted as a group‐composition effect rather than as direct evidence of individual behaviuor. Future work using individual GPS tracking will be needed to disentangle these group‐level patterns and precisely assess sex‐specific ranging behaviours and the effects of reproductive state.

Across three resource metrics—heterogeneity, patchiness, and inter‐patch spacing (dispersion of resource), we found no evidence that mean resource structure changed with season. This suggests a broadly stable foraging landscape for free‐ranging dogs across our sampling window, consistent with an urban–peri‐urban system where human‐derived inputs buffer seasonal fluctuations. The notable exception was variance in heterogeneity: allowing season‐specific dispersion greatly improved model fit, with lower dispersion in the post‐mating season despite unchanged means. Ecologically, this points to tighter clustering of heterogeneity values (i.e., more predictable resource mosaics) post‐mating, even though the average level of heterogeneity did not shift. Such stabilization could arise from routine human activities (e.g., steady waste generation) or seasonal events ending (like mating‐related movement across territories), reducing extremes in site‐to‐site variability.

Taken together, these findings underscore the complex interplay between resource availability and social dynamics in shaping the spatial organization of free‐ranging dog populations. The stability of group size, despite variations in resource availability, sex ratio, and territory size, suggests that social factors, such as dominance hierarchies, competition for mates, and the benefits of cooperative breeding (Paul et al. 2014b; Paul and Bhadra 2018), play a crucial role in constraining group size. This is consistent with the previous work (Valeix et al. 2012; Nel et al. 2013), which highlighted the role of social factors, such as intra‐group competition and the costs and benefits of group living, in constraining group size. Further research is needed to tease apart the relative importance of these social factors in different resource contexts and to fully elucidate their interplay in shaping the spatial ecology of free‐ranging dogs.

Their intimate association with humans exposes free‐ranging dogs to the full spectrum of human behaviour (Bhattacharjee et al. 2018; Bhattacharjee et al. 2021). While positive interactions, such as deliberate feeding and active begging from humans, represent a significant source of sustenance for these dogs (Bhattacharjee et al. 2018; Bhattacharjee et al. 2021), this reliance on human provisioning also makes them vulnerable to negative human actions. Sadly, humans are a major cause of high early life mortality in free‐ranging dogs (Paul et al. 2016).

The implications of these findings for dog population management and disease control are significant. In urban areas, where dog densities are high and resources are concentrated, targeted interventions may be needed to manage populations and reduce the risk of disease transmission. This could involve strategies such as spay/neuter programs, public education campaigns promoting responsible feeding practices, and improved waste management initiatives to limit the availability of anthropogenic food sources. In contrast, management efforts in rural areas may need to focus on different challenges, such as reducing conflict with livestock and wildlife through community‐based dog vaccination programs.

While the RDH provides a strong framework for interpreting our results, it is important to acknowledge that other factors, such as social structure, inter‐group competition, and human intervention, could also contribute to the observed patterns. Future research should aim to disentangle the relative importance of these factors in shaping the spatial ecology of free‐ranging dogs.

## Author Contributions

**Sourabh Biswas:** conceptualization (equal), data curation (lead), formal analysis (lead), investigation (lead), methodology (equal), project administration (lead), visualization (lead), writing – original draft (lead). **Kalyan Ghosh:** data curation (equal), investigation (supporting). **Sumedha Touhid:** project administration (supporting). **Srijaya Nandi:** investigation (equal), project administration (equal). **Arpan Bhattacharyya:** investigation (equal), project administration (equal). **Arunima Bhattacharyya:** investigation (supporting). **Milisha Das:** investigation (supporting). **Raktim Paul:** investigation (supporting). **Anindita Bhadra:** conceptualization (lead), funding acquisition (lead), methodology (lead), project administration (lead), resources (lead), supervision (lead), validation (equal), writing – review and editing (lead).

## Funding

Sourabh Biswas gratefully acknowledges the University Grants Commission (UGC), India, for providing a doctoral fellowship. This project was partially supported by the Janaki Ammal Award grant [BT/HRD/NBA‐NWB/39/2020‐21 (YC‐1)] awarded to Anindita Bhadra by the Department of Biotechnology (DBT), Government of India. Srijaya Nandi acknowledges support from the Department of Science and Technology—Innovation in Science Pursuit for Inspired Research (DST‐INSPIRE) program. Arpan Bhattacharyya is supported by the Council of Scientific and Industrial Research (CSIR), India.

## Disclosure

Statement on inclusion: Our study was conceptualized, conducted, and led entirely by researchers based in India, where all fieldwork was carried out. All authors are affiliated with academic institutions in the region, and several are local to the areas where the research took place. The study design, data collection, and interpretation were informed by local ecological knowledge and socio‐cultural context, ensuring that the research remained grounded in regional realities. We actively collaborated with local stakeholders throughout the study, particularly in efforts to reduce human–dog conflict through improved population management. Findings from this work are being shared with municipal authorities and local animal shelters to support evidence‐based interventions for free‐ranging dog control and conflict mitigation in urban areas.

## Conflicts of Interest

The authors declare no conflicts of interest.

## Supporting information


**Data S1:** ece372696‐sup‐0001‐DataS1.docx.

## Data Availability

The data for this manuscript is available at: https://data.mendeley.com/datasets/9zvpm6zrj9/2 (see Biswas, Ghosh, Touhid, et al. 2025).

## Appendix A

**TABLE A1:** Impact of season on the territory size of free‐ranging dog group.

Term	Estimate	Std. Error	*z* value	Pr(>z)
(Intercept)	0.33	0.18	1.84	> 0.05
Season: Post‐mating	−0.16	0.06	−2.59	< 0.05
Season: Pre‐mating	−0.04	0.06	−0.70	0.48

*Note:* GLMM formula = Territory size ~ Season (Pre‐mating/Mating/Post‐mating) + Group identity as random factor.

**TABLE A2:** Impact of season on the Group size of free‐ranging dog group.

Term	Estimate	Std. Error	*t* value	Pr(>z)
(Intercept)	1.82	0.07	25.63	< 0.0001
Season: Post‐mating	−0.04	0.04	−0.97	−0.33
Season: Pre‐mating	−0.04	0.05	−1.89	−0.06

*Note:* GLM formula = Group size ~ Season (Pre‐mating/Mating/Post‐mating) + Group identity as random factor.

## References

[ece372696-bib-0001] Alatalo, R. V. , A. Lundberg , and C. Glynn . 1986. “Female Pied Flycatchers Choose Territory Quality and Not Male Characteristics.” Nature 323, no. 6084: 152–153.

[ece372696-bib-0002] Baddeley, A. , and R. Turner . 2005. “spatstat: An R Package for Analyzing Spatial Point Patterns.” Journal of Statistical Software 12, no. 6: 1–42.

[ece372696-bib-0003] Bates, D. , M. Mächler , B. M. Bolker , and S. C. Walker . 2015. “Fitting Linear Mixed‐Effects Models Using lme4.” Journal of Statistical Software 67, no. 1: 1–48.

[ece372696-bib-0004] Bergström, A. , L. Frantz , R. Schmidt , et al. 2020. “Origins and Genetic Legacy of Prehistoric Dogs.” Science 370, no. 6516: 557–564. 33122379 10.1126/science.aba9572 PMC7116352

[ece372696-bib-0005] Bhadra, A. , D. Bhattacharjee , M. Paul , et al. 2016. “The Meat of the Matter: A Rule of Thumb for Scavenging Dogs?” Ethology Ecology and Evolution 28, no. 4: 427–440.

[ece372696-bib-0006] Bhattacharjee, D. , and A. Bhadra . 2021. “Response to Short‐Lived Human Overcrowding by Free‐Ranging Dogs.” Behavioral Ecology and Sociobiology 75, no. 7: 111.

[ece372696-bib-0007] Bhattacharjee, D. , R. Sarkar , S. Sau , and A. Bhadra . 2021. “Sociability of Indian Free‐Ranging Dogs ( *Canis lupus familiaris* ) Varies With Human Movement in Urban Areas.” Journal of Comparative Psychology 135, no. 1: 89–97. 32584054 10.1037/com0000241

[ece372696-bib-0008] Bhattacharjee, D. , S. Sau , and A. Bhadra . 2018. “Free‐Ranging Dogs Understand Human Intentions and Adjust Their Behavioral Responses Accordingly.” Frontiers in Ecology and Evolution 21: 6.

[ece372696-bib-0009] Biswas, S. , T. Bhowmik , K. Ghosh , et al. 2024. “Scavengers in the Human‐Dominated Landscape: An Experimental Study.” Philosophical Transactions of the Royal Society, B: Biological Sciences 379, no. 1909: 179. 10.1098/rstb.2023.0179. PMC11293862 39034699

[ece372696-bib-0010] Biswas, S. , K. Ghosh , H. Gope , and A. Bhadra . 2024. “Fair Game: Urban Free‐Ranging Dogs Balance Resource Use and Risk Aversion at Seasonal Fairs.” arXiv preprint arXiv:240617004. 10.1093/cz/zoaf030 PMC13045819 41940275

[ece372696-bib-0011] Biswas, S. , K. Ghosh , H. Gope , and A. Bhadra . 2025. “Fair Game: Urban Free‐Ranging Dogs Balance Resource Use and Risk Aversion at Seasonal Fairs.” Current Zoology. 10.1093/cz/zoaf030 PMC13045819 41940275

[ece372696-bib-0012] Biswas, S. , K. Ghosh , S. Touhid , et al. 2025. “Territory and Group Structure of Free‐Ranging Dogs in Urban–Rural India: Data for Testing the Resource Dispersion Hypothesis.” *Mendeley Data*. V2. 10.17632/9zvpm6zrj9.2.

[ece372696-bib-0013] Bivand, R. S. , E. Pebesma , and V. Gómez‐Rubio . 2013. Applied Spatial Data Analysis With R. Springer New York.

[ece372696-bib-0014] Bolker, B. 2019. “Getting Started With the glmmTMB Package.” Cran R‐Project Vignette. 9.

[ece372696-bib-0015] Champely, S. , C. Ekstrom , P. Dalgaard , et al. 2020. “pwr: Basic Functions for Power Analysis.” https://github.com/heliosdrm/pwr.

[ece372696-bib-0016] Clutton‐Brock, J. 1995. “Origins of the Dog: Domestication and Early History.” In The Domestic Dog: Its Evolution, Behaviour and Interactions With People, edited by J. Serpell . Cambridge University Press.

[ece372696-bib-0017] Clutton‐Brock, T. H. , F. E. Guinness , and S. D. Albon . 1982. Red Deer: Behavior and Ecology of Two Sexes. University of Chicago Press.

[ece372696-bib-0018] Creel, S. , M. G. L. Mills , and J. W. McNutt . 2004. “Demography and Population Dynamics of African Wild Dogs in Three Critical Populations.” Biology and Conservation of Wild Canids 12: 337–350.

[ece372696-bib-0019] David, M. L. 1970. The Wolf: The Ecology and Behavior of an Endangered Species. Natural History Press Garden City.

[ece372696-bib-0020] Dietz, J. M. 1984. Ecology and Social Organization of the Maned Wolf ( *Chrysocyon brachyurus* ). Smithsonian Institution.

[ece372696-bib-0021] Doncaster, C. P. , and D. W. Macdonald . 1997. “Activity Patterns and Interactions of Red Foxes ( *Vulpes vulpes* ) in Oxford City.” Journal of Zoology 241, no. 1: 73–87.

[ece372696-bib-0022] Erlandsson, R. , M. Hasselgren , K. Norén , D. Macdonald , and A. Angerbjörn . 2022. “Resources and Predation: Drivers of Sociality in a Cyclic Mesopredator.” Oecologia 198, no. 2: 381–392. 35112174 10.1007/s00442-022-05107-w PMC8858920

[ece372696-bib-0023] Hartig, F. , and M. F. Hartig . 2022. “Package ‘dharma’.” R Package. https://CRANR‐projectorg/package=DHARMa.

[ece372696-bib-0024] Jenner, N. , J. Groombridge , and S. M. Funk . 2011. “Commuting, Territoriality and Variation in Group and Territory Size in a Black‐Backed Jackal Population Reliant on a Clumped, Abundant Food Resource in Namibia.” Journal of Zoology 284, no. 4: 231–238.

[ece372696-bib-0025] Kamler, J. F. , and D. W. Macdonald . 2014. “Social Organization, Survival, and Dispersal of Cape Foxes ( *Vulpes chama* ) in South Africa.” Mammalian Biology 79, no. 1: 64–70.

[ece372696-bib-0026] Kittle, A. M. , M. Anderson , T. Avgar , et al. 2015. “Wolves Adapt Territory Size, Not Pack Size to Local Habitat Quality.” Journal of Animal Ecology 84, no. 5: 1177–1186. 25757794 10.1111/1365-2656.12366

[ece372696-bib-0027] Kruuk, H. 1985. “Group Territories of Carnivores: Empires and Enclaves.” Behavioural Ecology 25: 521–536.

[ece372696-bib-0028] Kruuk, H. , and R. Hewson . 1978. “Spacing and Foraging of Otters ( *Lutra lutra* ) in a Marine Habitat.” Journal of Zoology 185, no. 2: 205–212.

[ece372696-bib-0029] Kruuk, H. , A. Moorhouse , J. W. H. Conroy , L. Durbin , and S. Frears . 1989. “An Estimate of Numbers and Habitat Preferences of Otters *Lutra lutra* in Shetland, UK.” Biological Conservation 49, no. 4: 241–254.

[ece372696-bib-0030] Lüdecke, D. , M. Ben‐Shachar , I. Patil , P. Waggoner , and D. Makowski . 2021. “Performance: An R Package for Assessment, Comparison and Testing of Statistical Models.” Journal of Open Source Software 6, no. 60: 3139.

[ece372696-bib-0031] Macdonald, D. W. 1983. “The Ecology of Carnivore Social Behaviour.” Nature 301, no. 5899: 379–384.

[ece372696-bib-0032] Marneweck, C. , D. G. Marneweck , O. L. van Schalkwyk , G. Beverley , H. T. Davies‐Mostert , and D. M. Parker . 2019. “Spatial Partitioning by a Subordinate Carnivore Is Mediated by Conspecific Overlap.” Oecologia 191, no. 3: 531–540. 31535256 10.1007/s00442-019-04512-y

[ece372696-bib-0033] Mitani, J. C. , and P. S. Rodman . 1979. “Territoriality: The Relation of Ranging Pattern and Home Range Size to Defendability, With an Analysis of Territoriality Among Primate Species.” Behavioral Ecology and Sociobiology 5: 241–251.

[ece372696-bib-0034] Nel, J. A. J. , R. J. Loutit , R. Braby , and M. J. Somers . 2013. “Resource Dispersion, Territory Size and Group Size of Black‐Backed Jackals on a Desert Coast.” Acta Theriologica (Warsz) 58: 189–197.

[ece372696-bib-0035] Paul, M. , and A. Bhadra . 2018. “The Great Indian Joint Families of Free‐Ranging Dogs.” PLoS One 13, no. 5: e0197328. 29771960 10.1371/journal.pone.0197328 PMC5957358

[ece372696-bib-0036] Paul, M. , M. S. Sen , and A. Bhadra . 2014a. “Selfish Mothers? An Empirical Test of Parent‐Offspring Conflict Over Extended Parental Care.” Behavioural Processes 103: 17–22. 24216083 10.1016/j.beproc.2013.10.006

[ece372696-bib-0037] Paul, M. , M. S. Sen , and A. Bhadra . 2014b. “Grandmotherly Care: A Case Study in Indian Free‐Ranging Dogs.” Journal of Ethology 32, no. 2: 75–82.

[ece372696-bib-0038] Paul, M. , S. Sen Majumder , S. Sau , A. K. Nandi , and A. Bhadra . 2016. “High Early Life Mortality in Free‐Ranging Dogs Is Largely Influenced by Humans.” Scientific Reports 25: 6. 10.1038/srep19641 PMC4726281 26804633

[ece372696-bib-0039] Pebesma, E. , and R. Bivand . 2023. Spatial Data Science: With Applications in R. Chapman and Hall/CRC.

[ece372696-bib-0040] Pyke, G. H. 1979. “Optimal Foraging in Bumblebees: Rule of Movement Between Flowers Within Inflorescences.” Animal Behaviour 27: 1167–1181.

[ece372696-bib-0041] R Core Team . 2024. R: A Language and Environment for Statistical Computing. R Foundation for Statistical Computing. https://www.R‐project.org.

[ece372696-bib-0042] Ravi, S. 2023. “‘Urban/Rural’ India? What is ‘Urban/Rural’ India?” EAC‐PM Working Paper Series.

[ece372696-bib-0043] Sambo, M. , K. Hampson , J. Changalucha , et al. 2018. “Estimating the Size of Dog Populations in Tanzania to Inform Rabies Control.” Veterinary Sciences 5, no. 3: 77. 30205470 10.3390/vetsci5030077 PMC6164483

[ece372696-bib-0044] Sarkar, R. , and A. Bhadra . 2022. “How Do Animals Navigate the Urban Jungle? A Review of Cognition in Urban‐Adapted Animals.” Current Opinion in Behavioral Sciences 46: 101177.

[ece372696-bib-0045] Sarkar, R. , S. Sau , and A. Bhadra . 2019. “Scavengers Can Be Choosers: A Study on Food Preference in Free‐Ranging Dogs.” Applied Animal Behaviour Science 1, no. 216: 38–44.

[ece372696-bib-0046] Sen Majumder, S. , A. Bhadra , A. Ghosh , et al. 2014. “To Be or Not to Be Social: Foraging Associations of Free‐Ranging Dogs in an Urban Ecosystem.” Acta Ethologica 17, no. 1: 1–8.

[ece372696-bib-0047] Sen Majumder, S. , A. Chatterjee , and A. Bhadra . 2014. “A Dog's Day With Humans‐Time Activity Budget of Free‐Ranging Dogs in India.” Current Science 106: 874–878.

[ece372696-bib-0048] Serpell, J. 1995. The Domestic Dog: Its Evolution, Behaviour and Interactions With People. Cambridge University Press. https://books.google.co.in/books?id=I8HU_3ycrrEC.

[ece372696-bib-0049] Thanapongtharm, W. , S. Kasemsuwan , V. Wongphruksasoong , et al. 2021. “Spatial Distribution and Population Estimation of Dogs in Thailand: Implications for Rabies Prevention and Control.” Frontiers in Veterinary Science 8: 790701. 34993247 10.3389/fvets.2021.790701 PMC8724437

[ece372696-bib-0050] Travis, S. E. , and C. N. Slobodchikoff . 1993. “Effects of Food Resource Distribution on the Social System of Gunnison's Prairie Dog ( *Cynomys gunnisoni* ).” Canadian Journal of Zoology 71, no. 6: 1186–1192.

[ece372696-bib-0051] Travis, S. E. , C. N. Slobodchikoff , and P. Keim . 1995. “Ecological and Demographic Effects on Intraspecific Variation in the Social System of Prairie Dogs.” Ecology 76, no. 6: 1794–1803.

[ece372696-bib-0052] Valeix, M. , A. J. Loveridge , and D. W. Macdonald . 2012. “Influence of Prey Dispersion on Territory and Group Size of African Lions: A Test of the Resource Dispersion Hypothesis.” Ecology 93, no. 11: 2490–2496. 23236920 10.1890/12-0018.1

[ece372696-bib-0053] Valenzuela, D. , and D. W. Macdonald . 2002. “Home‐Range Use by White‐Nosed Coatis ( *Nasua narica* ): Limited Water and a Test of the Resource Dispersion Hypothesis.” Journal of Zoology 258, no. 2: 247–256.

[ece372696-bib-0054] Vangen, K. M. , J. Persson , A. Landa , R. Andersen , and P. Segerström . 2001. “Characteristics of Dispersal in Wolverines.” Canadian Journal of Zoology 79, no. 9: 1641–1649.

[ece372696-bib-0055] Warembourg, C. , G. Fournié , M. F. Abakar , et al. 2021. “Predictors of Free‐Roaming Domestic Dogs' Contact Network Centrality and Their Relevance for Rabies Control.” Scientific Reports 11, no. 1: 12898. 34145344 10.1038/s41598-021-92308-7 PMC8213792

[ece372696-bib-0056] Warret Rodrigues, C. , and J. D. Roth . 2023. “Coexistence of Two Sympatric Predators in a Transitional Ecosystem Under Constraining Environmental Conditions: A Perspective From Space and Habitat Use.” Movement Ecology 11, no. 1: 60. 37784160 10.1186/s40462-023-00421-1 PMC10544556

[ece372696-bib-0057] Wood, S. , and M. S. Wood . 2015. “Package ‘mgcv’.” R Package Version. 1(29), 729.

[ece372696-bib-0058] Zubiri, C. S. , and D. Gottelli . 1995. “Spatial Organization in the Ethiopian Wolf *Canis simensis* : Large Packs and Small Stable Home Ranges.” Journal of Zoology 237, no. 1: 65–81.

