# Supplementary material for: How Much Is Enough? An Empirical Test of the Resource Dispersion Hypothesis
Source: Ecol Evol. 2025 Dec 17;15(12):e72696. doi: 10.1002/ece3.72696 (PMC12711434; doi:10.1002/ece3.72696)

**Supplementary Materials**

**How Much is Enough? An Empirical Test of the Resource Dispersion Hypothesis**

***Table S1: Description of the study sites in different parts of West Bengal, India.***

| **Site name** | **Location type** | **Latitude** | **Longitude** |
| --- | --- | --- | --- |
| Alaipur | Rural | 88.474251 | 23.0118293 |
| Baduria | Rural | 88.8004816 | 22.72915 |
| Bally | Urban | 88.3383244 | 22.6494904 |
| Basai | Urban | 88.316486 | 22.6976315 |
| Belanagar | Urban | 88.3117332 | 22.6678121 |
| Belghoria | Urban | 88.3797398 | 22.666997 |
| Beltala | Rural | 88.3939025 | 23.2283944 |
| Belur | Urban | 88.3548006 | 22.633315 |
| Bhadreswar | Urban | 88.3455203 | 22.8276097 |
| Bongaon | Urban | 88.8283766 | 23.0467687 |
| Burdwan | Urban | 87.8612709 | 23.2328135 |
| Central Avenue | Urban | 88.3573682 | 22.5797246 |
| College Square | Urban | 88.3613361 | 22.5743423 |
| College Street | Urban | 88.3638454 | 22.5802029 |
| Dariapur | Rural | 87.8533322 | 21.798591 |
| Dhitara | Rural | 88.3324834 | 22.8331442 |
| Dunlop | Urban | 88.3784223 | 22.6519451 |
| Durgapur | Urban | 87.3115037 | 23.5207142 |
| Gangajoara | Rural | 88.4426879 | 22.4664808 |
| Garia | Urban | 88.392316 | 22.4659325 |
| Gayeshpur | Urban | 88.4958601 | 22.9555719 |
| Hindmotor | Urban | 88.3359927 | 22.6856585 |
| Hotar | Rural | 88.3976857 | 22.3075116 |
| Jhinuk Math | Rural | 88.5383401 | 22.9422634 |
| KalichaK | Rural | 87.6591729 | 22.6049378 |
| Kalyani | Urban | 88.4336533 | 22.974999 |
| Kalyani Simanta | Urban | 88.4296364 | 22.9894246 |
| Kalyannagar | Rural | 88.4869659 | 23.0067324 |
| Kanaipur | Rural | 88.3277284 | 22.7016015 |
| Konnagar | Urban | 88.3180873 | 22.7003518 |
| Majhdia | Rural | 88.7255001 | 23.4184776 |
| Majherchar | Rural | 88.4136343 | 22.984682 |
| Mandal Para | Rural | 88.3906401 | 22.637125 |
| Naihati | Urban | 88.4208212 | 22.88866 |
| Narenga | Rural | 87.963463 | 23.6346908 |
| Nawpara | Rural | 88.3906554 | 22.6423343 |
| Panihati | Urban | 88.4035205 | 22.6941993 |
| Patuli | Urban | 88.3842841 | 22.4714583 |
| Palashi | Rural | 88.260163 | 23.783412 |
| Raghunathpur | Rural | 88.3127139 | 22.6691818 |
| Rishra | Urban | 88.344105 | 22.7245565 |
| Serampor | Urban | 88.3404951 | 22.7505302 |
| Shalidaha | Rural | 88.4862612 | 22.8956445 |
| Sheoraphuli | Urban | 88.3220286 | 22.7707139 |
| Shibdaspur | Rural | 88.4813259 | 22.9074648 |
| Simhat | Rural | 88.5489917 | 22.9560819 |
| Sodepur | Urban | 88.3902671 | 22.6992269 |
| Uttarpara | Urban | 88.3349492 | 22.670397 |


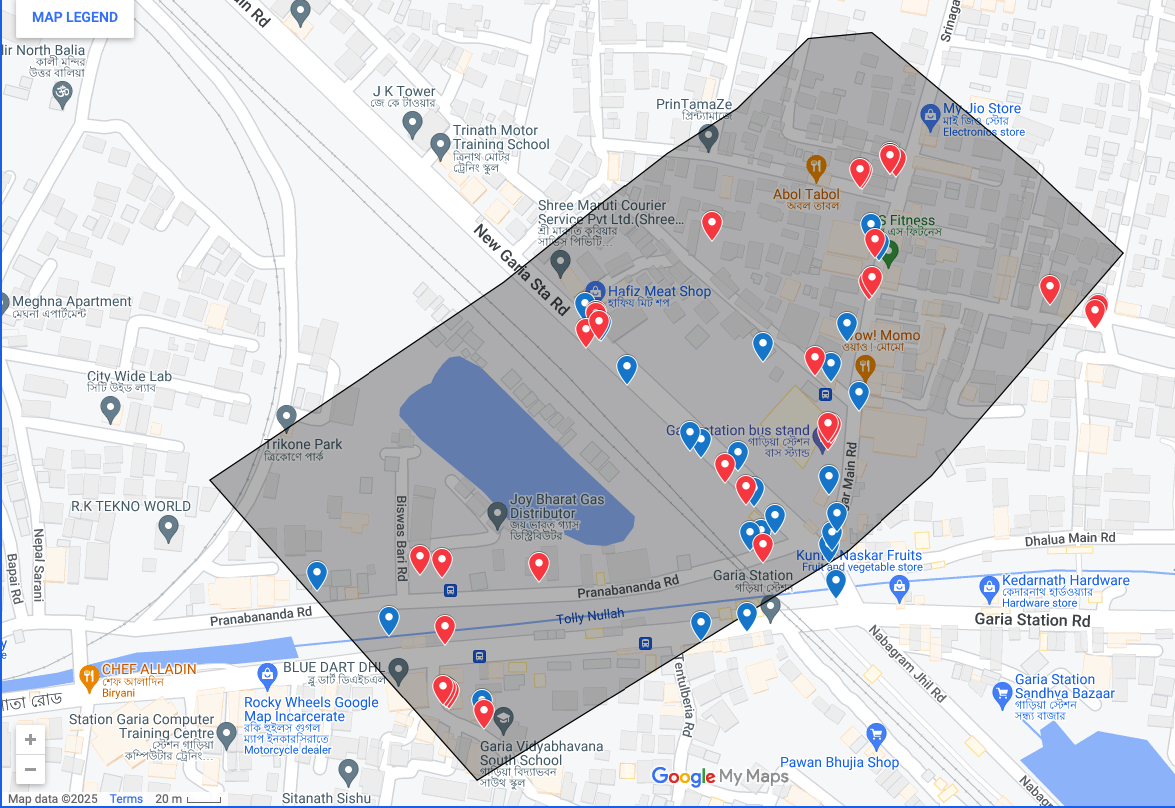


***Figure S1. Spatial distribution of dogs and resources within a sampled urban polygon.***

*This Google My Maps© image illustrates the spatial distribution of free-ranging dogs and resource points within a representative urban polygon. Red balloons indicate the locations of individual dogs observed during the census, while blue balloons represent identified resource points within the polygon.*

*
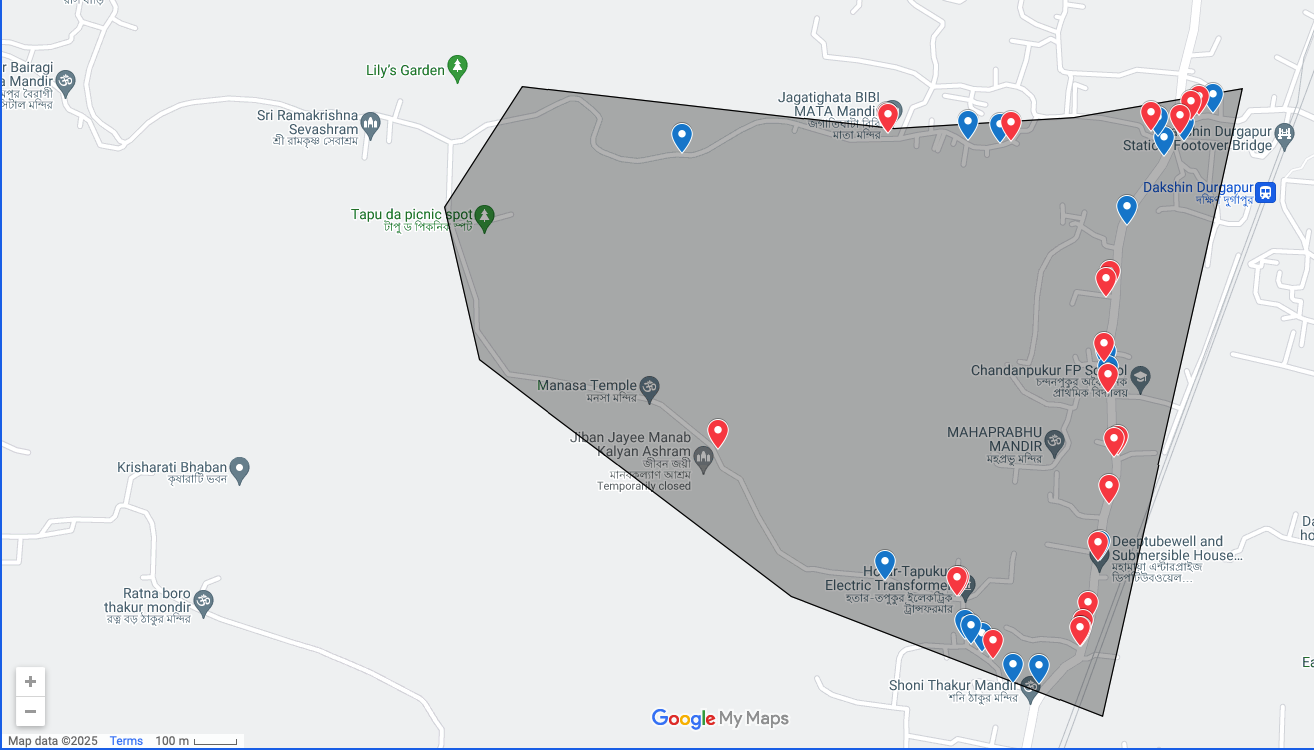
*

***Figure S2. Spatial distribution of dogs and resources within a sampled rural polygon.***

*This Google My Maps© image illustrates the spatial distribution of free-ranging dogs and resource points within a representative rural polygon. Red balloons indicate the locations of individual dogs observed during the census, while blue balloons represent identified resource points within the polygon.*

**R Codes and Model Diagnostics**

###### RDH_ Supplementary

rdh= read.csv("rdh.csv", stringsAsFactors = TRUE)

# Data strucutre
rdh_clean <- rdh[!is.na(rdh$ter_new), ]
rdh_clean2 <- rdh_clean[!is.na(rdh_clean$avg_dist), ]
########## Packages ##########
library(mgcv) # GAMs

## Loading required package: nlme

## This is mgcv 1.8-40. For overview type 'help("mgcv-package")'.

library(pscl) # pR2() for GLMs

## Classes and Methods for R developed in the
## Political Science Computational Laboratory
## Department of Political Science
## Stanford University
## Simon Jackman
## hurdle and zeroinfl functions by Achim Zeileis

library(performance) # diagnostics, VIF, etc.
library(DHARMa) # residual diagnostics

## This is DHARMa 0.4.6. For overview type '?DHARMa'. For recent changes, type news(package = 'DHARMa')

########## Data alias & sanity checks ##########
stopifnot(exists("rdh_clean2"))
d <- rdh_clean2

vars_all <- c("ter_new","dg_total","het_g","patch_r","mf_ratio","avg_dist")
stopifnot(all(vars_all %in% names(d)))

N_all <- nrow(d)
N_cc_size <- sum(complete.cases(d[, c("ter_new","dg_total","het_g","patch_r","mf_ratio","avg_dist")]))
N_cc_dogs <- sum(complete.cases(d[, c("dg_total","ter_new","het_g","patch_r","mf_ratio","avg_dist")]))
cat("Rows total:", N_all, "\n",
 "Complete cases (territory model):", N_cc_size, "\n",
 "Complete cases (dog model):", N_cc_dogs, "\n")

## Rows total: 36
## Complete cases (territory model): 36
## Complete cases (dog model): 36

########## GLMs (primary specifications) ##########
# Model 1: Territory size
m_size_glm <- glm(
 ter_new ~ dg_total + het_g + patch_r + mf_ratio + avg_dist,
 data = d, family = Gamma(link = "log")
)

# Model 2: Dog count
m_dogs_glm <- glm(
 dg_total ~ ter_new + het_g + patch_r + mf_ratio + avg_dist,
 data = d, family = Gamma(link = "log")
)

cat("\n=== GLM summaries ===\n")

##
## === GLM summaries ===

print(summary(m_size_glm))

##
## Call:
## glm(formula = ter_new ~ dg_total + het_g + patch_r + mf_ratio +
## avg_dist, family = Gamma(link = "log"), data = d)
##
## Deviance Residuals:
## Min 1Q Median 3Q Max
## -1.24645 -0.44140 -0.04427 0.39737 0.85250
##
## Coefficients:
## Estimate Std. Error t value Pr(>|t|)
## (Intercept) -2.7875005 0.5141320 -5.422 7.86e-06 ***
## dg_total -0.0005703 0.0499686 -0.011 0.99097
## het_g 0.1021408 0.0492026 2.076 0.04687 *
## patch_r 0.0411817 0.0125918 3.271 0.00277 **
## mf_ratio 0.3914299 0.1877555 2.085 0.04600 *
## avg_dist 0.0205599 0.0030506 6.740 2.14e-07 ***
## ---
## Signif. codes: 0 '***' 0.001 '**' 0.01 '*' 0.05 '.' 0.1 ' ' 1
##
## (Dispersion parameter for Gamma family taken to be 0.3767706)
##
## Null deviance: 32.330 on 34 degrees of freedom
## Residual deviance: 10.388 on 29 degrees of freedom
## AIC: 74.983
##
## Number of Fisher Scoring iterations: 8

print(summary(m_dogs_glm))

##
## Call:
## glm(formula = dg_total ~ ter_new + het_g + patch_r + mf_ratio +
## avg_dist, family = Gamma(link = "log"), data = d)
##
## Deviance Residuals:
## Min 1Q Median 3Q Max
## -0.75872 -0.19954 0.00145 0.14535 0.51570
##
## Coefficients:
## Estimate Std. Error t value Pr(>|t|)
## (Intercept) 1.776388 0.281387 6.313 6.77e-07 ***
## ter_new -0.027491 0.060977 -0.451 0.655
## het_g -0.003097 0.029149 -0.106 0.916
## patch_r 0.002921 0.007508 0.389 0.700
## mf_ratio 0.146842 0.114839 1.279 0.211
## avg_dist -0.001658 0.002528 -0.656 0.517
## ---
## Signif. codes: 0 '***' 0.001 '**' 0.01 '*' 0.05 '.' 0.1 ' ' 1
##
## (Dispersion parameter for Gamma family taken to be 0.09078836)
##
## Null deviance: 3.2721 on 34 degrees of freedom
## Residual deviance: 2.9227 on 29 degrees of freedom
## AIC: 152.64
##
## Number of Fisher Scoring iterations: 5

########## Nonlinearity screening with GAMs ##########
# Territory model: earlier results suggested only mild curvature in avg_dist
m_size_gam1 <- gam(
 ter_new ~ dg_total + het_g + patch_r + mf_ratio + s(avg_dist, k = 6),
 data = d, family = Gamma(link="log"), method="REML", select=TRUE
)

# Dog model: penalized GAM with small k per term (strong improvement in your test)
m_dogs_gam2 <- gam(
 dg_total ~ s(ter_new, k = 4, bs = "cr") +
 s(het_g, k = 4, bs = "cr") +
 s(patch_r, k = 4, bs = "cr") +
 s(mf_ratio,k = 4, bs = "cr") +
 s(avg_dist,k = 4, bs = "cr"),
 data = d, family = Gamma(link="log"), method="REML", select=TRUE
)

cat("\n=== AIC & AICc comparisons ===\n")

##
## === AIC & AICc comparisons ===

print(AIC(m_size_glm, m_size_gam1))

## df AIC
## m_size_glm 7.000000 87.03606
## m_size_gam1 8.756833 78.49802

print(AIC(m_dogs_glm, m_dogs_gam2))

## df AIC
## m_dogs_glm 7.000000 156.1486
## m_dogs_gam2 3.996285 149.5233

cat("\n=== GAM smooth tables ===\n")

##
## === GAM smooth tables ===

cat("\nTerritory model (GAM with s(avg_dist)):\n"); print(summary(m_size_gam1)$s.table)

##
## Territory model (GAM with s(avg_dist)):

## edf Ref.df F p-value
## s(avg_dist) 2.295605 5 10.72641 0

cat("\nDog model (penalized GAM with k=4 per term):\n"); print(summary(m_dogs_gam2)$s.table)

##
## Dog model (penalized GAM with k=4 per term):

## edf Ref.df F p-value
## s(ter_new) 1.475719e-05 3 1.287616e-06 0.7091997
## s(het_g) 5.318895e-05 3 1.203531e-05 0.4557058
## s(patch_r) 1.116583e-05 3 1.673267e-07 0.9706369
## s(mf_ratio) 8.298470e-01 3 6.826085e-01 0.1025906
## s(avg_dist) 4.727102e-01 3 2.898700e-01 0.1802813

########## Optional: interaction check (biologically motivated) ##########
# Keep disabled by default; uncomment if you want to test
# m_size_glm_int <- update(m_size_glm, . ~ . + avg_dist:mf_ratio + avg_dist:het_g)
# m_dogs_glm_int <- update(m_dogs_glm, . ~ . + avg_dist:mf_ratio + avg_dist:het_g)
# cat("\n=== AIC with interactions (optional) ===\n")
# print(AIC(m_size_glm, m_size_glm_int))
# print(AIC(m_dogs_glm, m_dogs_glm_int))

########## Performance metrics ##########
cat("\n=== Pseudo-R2 for GLMs (McFadden, Cox-Snell, Nagelkerke) ===\n")

##
## === Pseudo-R2 for GLMs (McFadden, Cox-Snell, Nagelkerke) ===

print(pscl::pR2(m_size_glm))

## fitting null model for pseudo-r2
## llh llhNull G2 McFadden r2ML r2CU
## -36.5180293 -53.4296322 33.8232058 0.3165210 0.6091899 0.6421921

print(pscl::pR2(m_dogs_glm))

## fitting null model for pseudo-r2
## llh llhNull G2 McFadden r2ML r2CU
## -71.07431232 -72.85676493 3.56490522 0.02446516 0.09428007 0.09595587

cat("\n=== Model diagnostics & checks ===\n")

##
## === Model diagnostics & checks ===

# Collinearity (VIF-like)
print(performance::check_collinearity(m_size_glm))

## # Check for Multicollinearity
##
## Low Correlation
##
## Term VIF Increased SE Tolerance
## dg_total 1.06 1.03 0.94
## het_g 1.52 1.23 0.66
## patch_r 1.54 1.24 0.65
## mf_ratio 1.11 1.05 0.90
## avg_dist 1.09 1.04 0.92

print(performance::check_collinearity(m_dogs_glm))

## # Check for Multicollinearity
##
## Low Correlation
##
## Term VIF Increased SE Tolerance
## ter_new 2.07 1.44 0.48
## het_g 1.60 1.26 0.63
## patch_r 1.86 1.36 0.54
## mf_ratio 1.42 1.19 0.70
## avg_dist 1.71 1.31 0.58

# Residual diagnostics (GLMs)
cat("\nDHARMa residuals: territory GLM\n")

##
## DHARMa residuals: territory GLM

plot(simulateResiduals(m_size_glm))

## Unable to calculate quantile regression for quantile 0.25. Possibly to few (unique) data points / predictions. Will be ommited in plots and significance calculations.

## Unable to calculate quantile regression for quantile 0.5. Possibly to few (unique) data points / predictions. Will be ommited in plots and significance calculations.

## Unable to calculate quantile regression for quantile 0.75. Possibly to few (unique) data points / predictions. Will be ommited in plots and significance calculations.


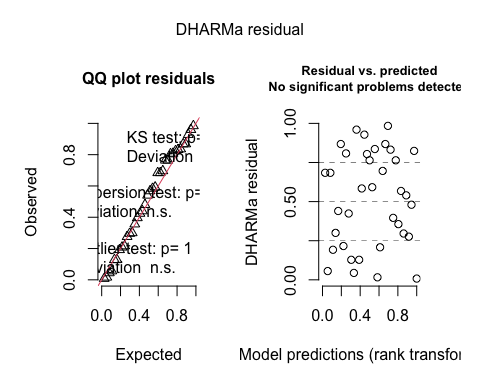


cat("\nDHARMa residuals: dog GLM\n")

##
## DHARMa residuals: dog GLM

plot(simulateResiduals(m_dogs_glm))

## Unable to calculate quantile regression for quantile 0.25. Possibly to few (unique) data points / predictions. Will be ommited in plots and significance calculations.

## Unable to calculate quantile regression for quantile 0.5. Possibly to few (unique) data points / predictions. Will be ommited in plots and significance calculations.

## Unable to calculate quantile regression for quantile 0.75. Possibly to few (unique) data points / predictions. Will be ommited in plots and significance calculations.


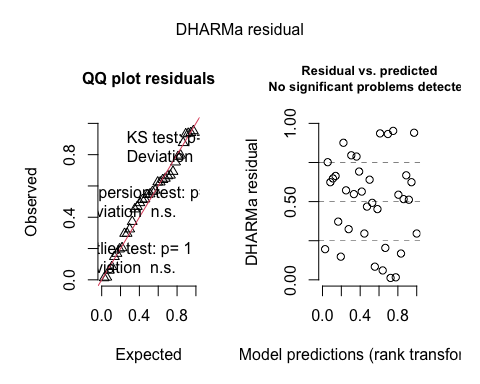


# Residual diagnostics (GAMs)
cat("\nGAM diagnostics: territory GAM\n")

##
## GAM diagnostics: territory GAM

gam.check(m_size_gam1)


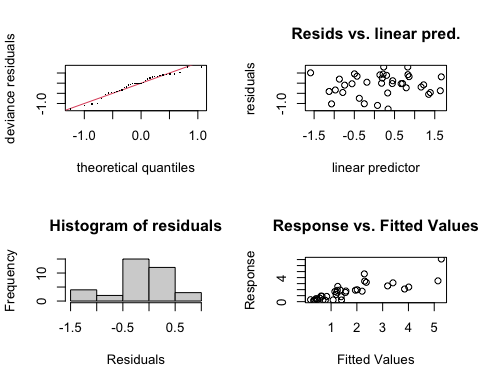


##
## Method: REML Optimizer: outer newton
## full convergence after 7 iterations.
## Gradient range [-1.517533e-07,4.083058e-08]
## (score 44.90036 & scale 0.2702539).
## Hessian positive definite, eigenvalue range [0.3021273,17.43582].
## Model rank = 10 / 10
##
## Basis dimension (k) checking results. Low p-value (k-index<1) may
## indicate that k is too low, especially if edf is close to k'.
##
## k' edf k-index p-value
## s(avg_dist) 5.0 2.3 1 0.53

cat("\nGAM diagnostics: dog GAM\n")

##
## GAM diagnostics: dog GAM

gam.check(m_dogs_gam2)


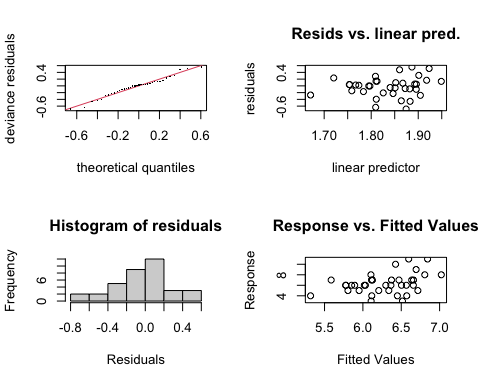


##
## Method: REML Optimizer: outer newton
## full convergence after 13 iterations.
## Gradient range [-4.071905e-06,2.058956e-05]
## (score 74.40172 & scale 0.08221205).
## Hessian positive definite, eigenvalue range [6.43064e-07,18.02322].
## Model rank = 16 / 16
##
## Basis dimension (k) checking results. Low p-value (k-index<1) may
## indicate that k is too low, especially if edf is close to k'.
##
## k' edf k-index p-value
## s(ter_new) 3.00e+00 1.48e-05 1.06 0.63
## s(het_g) 3.00e+00 5.32e-05 1.11 0.69
## s(patch_r) 3.00e+00 1.12e-05 1.11 0.71
## s(mf_ratio) 3.00e+00 8.30e-01 0.81 0.06 .
## s(avg_dist) 3.00e+00 4.73e-01 1.31 0.97
## ---
## Signif. codes: 0 '***' 0.001 '**' 0.01 '*' 0.05 '.' 0.1 ' ' 1

########## Likelihood-ratio tests vs null ##########
m_size_null <- glm(ter_new ~ 1, data = d, family = Gamma(link = "log"))
m_dogs_null <- glm(dg_total ~ 1, data = d, family = Gamma(link = "log"))

cat("\n=== Likelihood-ratio tests vs. null ===\n")

##
## === Likelihood-ratio tests vs. null ===

print(anova(m_size_null, m_size_glm, test="Chisq"))

## Analysis of Deviance Table
##
## Model 1: ter_new ~ 1
## Model 2: ter_new ~ dg_total + het_g + patch_r + mf_ratio + avg_dist
## Resid. Df Resid. Dev Df Deviance Pr(>Chi)
## 1 35 32.335
## 2 30 13.731 5 18.604 1.858e-09 ***
## ---
## Signif. codes: 0 '***' 0.001 '**' 0.01 '*' 0.05 '.' 0.1 ' ' 1

print(anova(m_dogs_null, m_dogs_glm, test="Chisq"))

## Analysis of Deviance Table
##
## Model 1: dg_total ~ 1
## Model 2: dg_total ~ ter_new + het_g + patch_r + mf_ratio + avg_dist
## Resid. Df Resid. Dev Df Deviance Pr(>Chi)
## 1 35 3.2746
## 2 30 2.9700 5 0.30454 0.6455

########## Partial-effect plots for GAMs (quick look) ##########
# (Comment out if running non-interactively)
par(mfrow=c(1,1))
plot(m_size_gam1, pages=1, shade=TRUE, scheme=1)


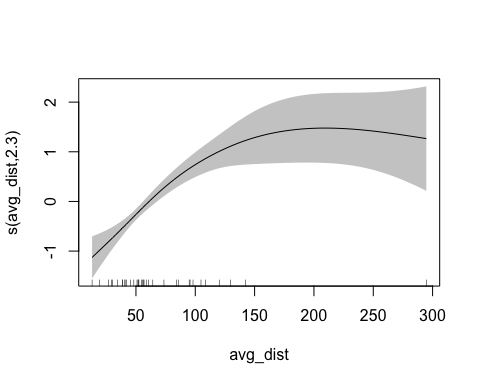


plot(m_dogs_gam2, pages=1, shade=TRUE, scheme=1)


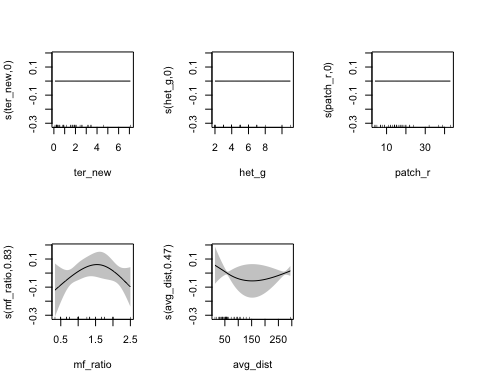


########## Compact reporting block (copy values to manuscript) ##########
cat("\n=== REPORTING BLOCK ===\n")

##
## === REPORTING BLOCK ===

# Territory model: GLM vs GAM (one smooth)
cat("Territory model: AIC(GLM) =", AIC(m_size_glm),
 "; AIC(GAM s(avg_dist)) =", AIC(m_size_gam1),
 "; ΔAIC =", AIC(m_size_glm) - AIC(m_size_gam1), "\n")

## Territory model: AIC(GLM) = 87.03606 ; AIC(GAM s(avg_dist)) = 78.49802 ; ΔAIC = 8.538036

cat("Territory GAM smooth EDF & p (avg_dist):\n")

## Territory GAM smooth EDF & p (avg_dist):

print(summary(m_size_gam1)$s.table)

## edf Ref.df F p-value
## s(avg_dist) 2.295605 5 10.72641 0

# Dog model: GLM vs penalized GAM
cat("Dog model: AIC(GLM) =", AIC(m_dogs_glm),
 "; AIC(GAM penalized) =", AIC(m_dogs_gam2),
 "; ΔAIC =", AIC(m_dogs_glm) - AIC(m_dogs_gam2), "\n")

## Dog model: AIC(GLM) = 156.1486 ; AIC(GAM penalized) = 149.5233 ; ΔAIC = 6.625277

cat("Dog GAM smooths (edf ~ 1 ≈ linear, >1 suggests curvature):\n")

## Dog GAM smooths (edf ~ 1 ≈ linear, >1 suggests curvature):

print(summary(m_dogs_gam2)$s.table)

## edf Ref.df F p-value
## s(ter_new) 1.475719e-05 3 1.287616e-06 0.7091997
## s(het_g) 5.318895e-05 3 1.203531e-05 0.4557058
## s(patch_r) 1.116583e-05 3 1.673267e-07 0.9706369
## s(mf_ratio) 8.298470e-01 3 6.826085e-01 0.1025906
## s(avg_dist) 4.727102e-01 3 2.898700e-01 0.1802813

########## (Optional) Save objects for later use ##########
# save(m_size_glm, m_dogs_glm, m_size_gam1, m_dogs_gam2, file = "rdh_models.RData")

########

**Association with resource matrices and season**

rdh= read.csv("rdh.csv", stringsAsFactors = TRUE)

# Data strucutre
rdh_clean <- rdh[!is.na(rdh$ter_new), ]

library(glmmTMB)

## Warning in checkDepPackageVersion(dep_pkg = "TMB"): Package version inconsistency detected.
## glmmTMB was built with TMB version 1.9.0
## Current TMB version is 1.9.1
## Please re-install glmmTMB from source or restore original 'TMB' package (see '?reinstalling' for more information)

### Resource heterogeneity

het_G_mod= glmmTMB(het_g ~ season +
 (1|grp_n),
 family = Gamma(link = "log"), data = rdh_clean)

m_disp <- glmmTMB(
 het_g ~ season + (1|grp_n),
 family = Gamma(link = "log"),
 dispformula = ~ season, # <- key change: season-specific dispersion
 data = rdh_clean)

summary(m_disp)

## Family: Gamma ( log )
## Formula: het_g ~ season + (1 | grp_n)
## Dispersion: ~season
## Data: rdh_clean
##
## AIC BIC logLik deviance df.resid
## 212.1 229.1 -99.1 198.1 77
##
## Random effects:
##
## Conditional model:
## Groups Name Variance Std.Dev.
## grp_n (Intercept) 0.1785 0.4225
## Number of obs: 84, groups: grp_n, 36
##
## Conditional model:
## Estimate Std. Error z value Pr(>|z|)
## (Intercept) 1.554963 0.071717 21.682 <2e-16 ***
## seasonPost mating -0.031453 0.025412 -1.238 0.216
## seasonPre mating 0.008398 0.007903 1.063 0.288
## ---
## Signif. codes: 0 '***' 0.001 '**' 0.01 '*' 0.05 '.' 0.1 ' ' 1
##
## Dispersion model:
## Estimate Std. Error z value Pr(>|z|)
## (Intercept) 6.804 1.197 5.683 1.33e-08 ***
## seasonPost mating -2.648 1.102 -2.403 0.0163 *
## seasonPre mating 1.438 6.549 0.220 0.8262
## ---
## Signif. codes: 0 '***' 0.001 '**' 0.01 '*' 0.05 '.' 0.1 ' ' 1

anova(het_G_mod, m_disp)

## Data: rdh_clean
## Models:
## het_G_mod: het_g ~ season + (1 | grp_n), zi=~0, disp=~1
## m_disp: het_g ~ season + (1 | grp_n), zi=~0, disp=~season
## Df AIC BIC logLik deviance Chisq Chi Df Pr(>Chisq)
## het_G_mod 5 246.40 258.56 -118.201 236.40
## m_disp 7 212.11 229.13 -99.055 198.11 38.292 2 4.841e-09 ***
## ---
## Signif. codes: 0 '***' 0.001 '**' 0.01 '*' 0.05 '.' 0.1 ' ' 1

library(DHARMa)

## This is DHARMa 0.4.6. For overview type '?DHARMa'. For recent changes, type news(package = 'DHARMa')

plot(simulateResiduals(m_disp))


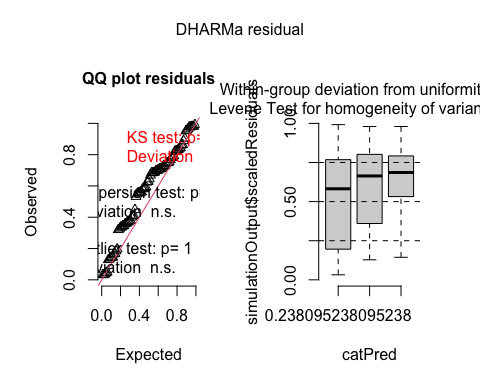


##### Resource Patchiness


patch_r_mod= glmmTMB(patch_r ~ season +
 (1|grp_n),
 family = Gamma(link = "log"), data = rdh_clean)

summary(patch_r_mod)

## Family: Gamma ( log )
## Formula: patch_r ~ season + (1 | grp_n)
## Data: rdh_clean
##
## AIC BIC logLik deviance df.resid
## 437.7 449.8 -213.8 427.7 79
##
## Random effects:
##
## Conditional model:
## Groups Name Variance Std.Dev.
## grp_n (Intercept) 0.2959 0.544
## Number of obs: 84, groups: grp_n, 36
##
## Dispersion estimate for Gamma family (sigma^2): 0.00575
##
## Conditional model:
## Estimate Std. Error z value Pr(>|z|)
## (Intercept) 2.659162 0.092044 28.890 <2e-16 ***
## seasonPost mating -0.018898 0.021044 -0.898 0.369
## seasonPre mating 0.002248 0.022467 0.100 0.920
## ---
## Signif. codes: 0 '***' 0.001 '**' 0.01 '*' 0.05 '.' 0.1 ' ' 1

library(DHARMa)
plot(simulateResiduals(patch_r_mod))


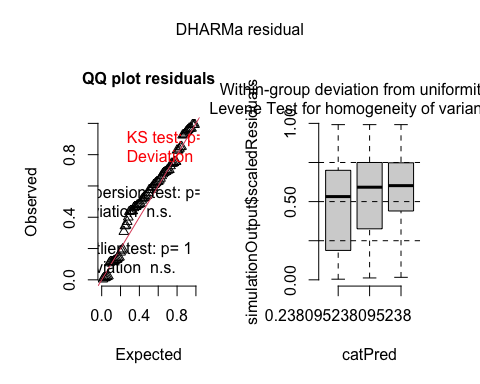


##### Resource dispersion

# Fit a standard linear model (no random effect)
disp_mod_lm <- lm(log(avg_dist) ~ season, data = rdh_clean)

# Now check the summary, which will be stable and interpretable
summary(disp_mod_lm)

##
## Call:
## lm(formula = log(avg_dist) ~ season, data = rdh_clean)
##
## Residuals:
## Min 1Q Median 3Q Max
## -1.56547 -0.30761 -0.01514 0.41872 1.67083
##
## Coefficients:
## Estimate Std. Error t value Pr(>|t|)
## (Intercept) 4.1334 0.1115 37.076 <2e-16 ***
## seasonPost mating -0.1183 0.1505 -0.786 0.434
## seasonPre mating -0.1685 0.1681 -1.002 0.319
## ---
## Signif. codes: 0 '***' 0.001 '**' 0.01 '*' 0.05 '.' 0.1 ' ' 1
##
## Residual standard error: 0.5899 on 81 degrees of freedom
## Multiple R-squared: 0.01362, Adjusted R-squared: -0.01074
## F-statistic: 0.559 on 2 and 81 DF, p-value: 0.5739

library(DHARMa)
plot(simulateResiduals(disp_mod_lm))


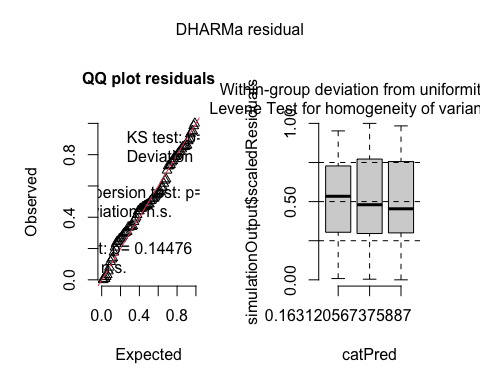

Supplement: Supplementary file 1 — Data S1: ece372696‐sup‐0001‐DataS1.docx. [file ECE3-15-e72696-s001.docx]
